# Benefits and harms of annual, biennial, or triennial breast cancer mammography screening for women at average risk of breast cancer: a systematic review for the European Commission Initiative on Breast Cancer (ECIBC)

**DOI:** 10.1038/s41416-021-01521-8

**Published:** 2021-11-26

**Authors:** Carlos Canelo-Aybar, Margarita Posso, Nadia Montero, Ivan Solà, Zuleika Saz-Parkinson, Stephen W. Duffy, Markus Follmann, Axel Gräwingholt, Paolo Giorgi Rossi, Pablo Alonso-Coello

**Affiliations:** 1 Iberoamerican Cochrane Centre - Department of Clinical Epidemiology and Public Health, Biomedical Research Institute Sant Pau (IIB Sant Pau), Barcelona, Spain; 2 CIBER de Epidemiología y Salud Pública (CIBERESP), Madrid, Spain; 3 Department of Paediatrics, Obstetrics and Gynaecology, Preventive Medicine, and Public Health. PhD Programme in Methodology of Biomedical Research and Public Health, Universitat Autònoma de Barcelona, Bellaterra, Barcelona, Spain; 4 Department of Epidemiology and Evaluation, IMIM (Hospital del Mar Medical Research Institute), Barcelona, Spain; 5 European Commission, Joint Research Centre (JRC), Ispra, Italy; 6 Wolfson Institute of Preventive Medicine, Queen Mary University of London, London, UK; 7 German Cancer Society, Berlin, Germany; 8 Radiologie am Theater, Paderborn, Germany; 9 Epidemiology Unit, Azienda Unità Sanitaria Locale – IRCCS di Reggio Emilia, Reggio Emilia, Italy

**Keywords:** Population screening, Breast cancer

## Abstract

**Background:**

Although mammography screening is recommended in most European countries, the balance between the benefits and harms of different screening intervals is still a matter of debate. This review informed the European Commission Initiative on Breast Cancer (BC) recommendations.

**Methods:**

We searched PubMed, EMBASE, and the Cochrane Library to identify RCTs, observational or modelling studies, comparing desirable (BC deaths averted, QALYs, BC stage, interval cancer) and undesirable (overdiagnosis, false positive related, radiation related) effects from annual, biennial, or triennial mammography screening in women of average risk for BC. We assessed the certainty of the evidence using the GRADE approach.

**Results:**

We included one RCT, 13 observational, and 11 modelling studies. In women 50–69, annual compared to biennial screening may have small additional benefits but an important increase in false positive results; triennial compared to biennial screening may have smaller benefits while avoiding some harms. In younger women (aged 45–49), annual compared to biennial screening had a smaller gain in benefits and larger harms, showing a less favourable balance in this age group than in women 50–69. In women 70–74, there were fewer additional harms and similar benefits with shorter screening intervals. The overall certainty of the evidence for each of these comparisons was very low.

**Conclusions:**

In women of average BC risk, screening intervals have different trade-offs for each age group. The balance probably favours biennial screening in women 50–69. In younger women, annual screening may have a less favourable balance, while in women aged 70–74 years longer screening intervals may be more favourable.

## Introduction

Breast cancer (BC) is the second most prevalent cancer in the world and the most frequent among women [1]. In the European Union, 404,920 women were diagnosed with BC and 98,755 women died during 2018 [2]. Despite these high rates, the mortality risk of BC has decreased over the last decades due to improvements in treatment, services quality, and to early diagnosis linked to the implementation of population-based screening programmes [3]. However, there is still ongoing research and debate on how to best implement BC screening programmes, including which is the optimal mammography screening interval.

Published recommendations on mammography screening frequencies vary among organisations. The National Health Service Breast Screening Program (NHSBSP) of the United Kingdom, recommends screening every 3 years to women aged 50–70 (47–73 in England) [4]. The United States Prevention Services Task Force (USPSTF) recommends biennial mammography for women aged 50–74 and making a case by case decision for women in their 40s [5]. The American Cancer Society recommends annual screening between the ages of 45 and 54 (with the option of starting annual screening between 40 and 44), and screening every two years from age 55 or continue annually if the woman is in good health and expected to live ten more years [6].

Previous studies have suggested that the balance between benefits and harms for different screening intervals might vary depending on the age subgroup. A modelling study found that for every 1000 women aged 50–74, biennial screening avoided seven BC deaths, while annual screening had similar benefits but caused more harms [7]. Observational data from the US Breast Cancer Surveillance Consortium (BCSC) registries, observed that premenopausal women undergoing biennial screening had more BC lesions with less favourable prognostic characteristics compared to those having annual screening [8].

In 2015, the European Commission Initiative on Breast Cancer (ECIBC) was launched to develop the European Guidelines on Breast Cancer Screening and Diagnosis. This article describes the systematic review that informed the recommendations about mammography screening intervals for women of average breast cancer risk in three separate age subgroups [9, 10]. During the guideline development process [9], the Guidelines Development Group (GDG) made detailed considerations about the balance between desirable and undesirable effects [9], values and preferences, equity, acceptability and feasibility; these considerations are described in the published methodology and summary of recommendations [9, 10] (https://healthcare-quality.jrc.ec.europa.eu/european-breast-cancer-guidelines/screening-ages-and-frequencies).

## Methods

### Structured question and outcome prioritisation

The clinical question ‘*Should an annual, biennial or triennial screening frequency be used for screening asymptomatic women*?’ was prioritised by the GDG (Box 1: Structured clinical question). This review focused on the three age subgroups for which the European Guidelines previously issued recommendations for screening (45–49, 50–69, and 70–74 years old). The GDG prioritised the outcomes using a 1–9 scale (7–9 critical; 4–6 important; 1–3 of limited importance) [11].

Box 1 PICO structured clinical question

**Table:** 

**Population**	**Intervention**	**Comparison**	**Outcomes**
Women who are at average risk of breast cancer: • 45–49 years • 50–69 years • 70–74 years	Annual, biennial or triennial mammography screening (film or digital)	Another interval (annual, biennial or triennial)	1. Breast cancer mortality 2. Incidence of interval cancer 3. Stage of breast cancer 4. Radiation induced breast cancers 5. Deaths due to radiation induced breast cancers 6. Quality of life 7. False positive related adverse effects 8. Overdiagnosis

### Data sources and searches

We initially searched MEDLINE (via PubMed, October 2016), EMBASE (via Ovid, October 2016) and CENTRAL (via The Cochrane Library, October 2016) databases using pre-defined algorithms for individual studies. We updated our initial search in MEDLINE (via PubMed) and EMBASE (via Ovid) in April 2020 (Supplementary Table S1*: Protocol Systematic Review*, Supplementary Table S2*: Search strategy*).

### Study selection

We included studies published in English of the following designs: (I) randomised clinical trials (RCTs), (ii) observational studies such as cohorts, time trend (before-after), or analysis of population surveillance registries, and (iii) decision analytic models (hereafter referred to as modelling studies) (Supplementary Tables S3a and 3b). All studies included at least two screening intervals in one of the age groups of interest; screening intervals from observational studies should had been defined based on at least two examinations prior to diagnosis; modelling studies should have assumed 100% adherence to the screening programmes and applied no discounting to the effects. Due to sparse empirical evidence in the 45–49 age subgroup, we included RCTs and observational studies that recruited women from 40 to 49.

We excluded studies of women at high risk for breast cancer, i.e. having known susceptibility gene mutations (BRCA1/BRCA2), a history of previous breast cancer or lobular neoplasia, exposure to chest irradiation (other than diagnostic imaging over that anatomical area) or having a direct family member with breast cancer.

Pairs of reviewers (CCA, MP), after calibration, assessed eligibility and reviewed the full text of the selected references. Discrepancies were resolved either by consensus or with the help of a third reviewer.

### Data extraction and risk of bias assessment

CCA and MP independently extracted details of the study design, patient population, setting, screening method, follow-up, mammography intervals and results. If needed, we requested additional data from the authors. We assessed the risk of bias (or credibility for modelling studies) with the following tools: (I) for RCTs with the Cochrane Risk of Bias Assessment tool [12] (ii) for observational studies with the Risk of Bias in Non-randomised Studies of Intervention (ROBINS-I) [13], (iii) for modelling studies with the Questionnaire to Assess Relevance and Credibility of Modelling Studies (the ISPOR-AMCP-NPC Good Practice Task Force) [14].

### Data analysis

We prioritised observational studies reporting the longest observation time when different studies used the same surveillance or clinical registries from an identical population covering overlapping time periods. We prioritised the more direct evidence for a European population of average risk when data was stratified by women´s characteristics (i.e. white women instead of other ethnic groups).

Modelling studies reported the incremental number of events for each screening interval compared to a non-screening scenario. For some studies, we calculated the number of events by subtracting overlapping age groups (i.e. to obtain events in annual screening in women 45– 49 years old, we subtracted the estimates in women 50–69 from the larger group of 45–69). We used the estimates for women with scattered fibroglandular breast density when they were only reported by breast density categories. Across the different studies, we presented the range of the absolute difference of events per each pairwise screening interval comparison.

We did not attempt to conduct a meta-analysis of relative risks (RR) or odds ratios (OR) from empirical studies because there were not enough studies across age groups to be meaningful or because several publications reported the same population data at overlapping time periods.

### Certainty of the evidence

We rated the certainty of the evidence, as high, moderate, low or very low, for each outcome based on the standard GRADE approach for RCTs and observational studies [15, 16]. To apply the GRADE approach to modelling studies, we considered the certainty would depart from the lowest certainty of the bodies of evidence that informed the main inputs in the model. We used the credibility and relevance items from the ISPOR-AMCP-NPC tool to inform the judgments for the risk of bias and indirectness domains.

As is customary in systematic reviews, we adopted a partially contextualised approach to rate the certainty of evidence, this means that for a point (or range) estimate of a single outcome we assessed our certainty that the true effects lie within the boundaries of what we consider a trivial, small, medium or large effect without considering the evidence from other outcomes [17]. During the development of recommendations, guideline panel members might consider our results using a contextualised approach which means considering the evidence from other critical outcomes (i.e. whether the benefits are consistent across outcomes) when rating the certainty for a single outcome [17].

## Results

### Search results

**Fig. 1:**
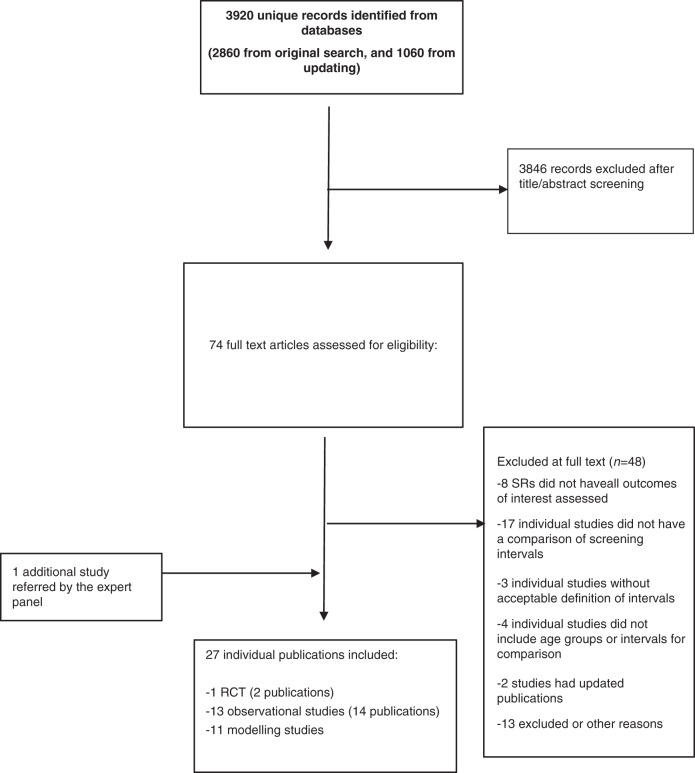
PRISMA flow diagram of literature search and selection.

We included 22 studies from 2860 unique citations in our initial evidence synthesis in October 2016 which was used to develop the ECIBC recommendations. After the updated search in April, 2020, we included 3 additional studies comprising a total of 25 studies (from 27 publications) during both periods: one RCT [18, 19], 11 modelling studies [7, 20–29] and 13 observational studies (Fig. 1) [8, 30–42]. The list of excluded studies and reasons for exclusion are described in Supplementary Table S4.

### Studies’ characteristics

We provide here a summary of the study design, and the main results for only the three age groups of interest. When there is empirical data (from observational or RCTs) we rely primarily on those estimates instead of simulated number of events from modelling studies. To interpret the modelling estimated events, we must consider that they represent the estimated events for a cohort of individuals from the time of screening until death or during the individual´s lifetime (or other given time point). The estimated 10-year probability of false positive or false biopsy recommendation in the observational studies were estimated using a previously described statistical model [43]. A detailed reporting of the results from studies covering larger age groups (i.e. 66–74 years) can be found in Supplementary Table S3a, b.

The only available RCT was conducted between 1989 and 1996, the United Kingdom Co-ordinating Committee on Cancer Research (UKCCCR) trial of Breast Screening Frequency and randomly allocated 99,389 women aged 50–62 to either annual or triennial screening [19]. Of the women originally invited to either of the screening arms, 38,492 (77%) attended triennial screening and 37,530 (76%) attended annual screening. The primary end point was predicted mortality based on two validated risk-models. However, as the UKCCR published observed data for survival up to the end of 2006, we reported these estimates in our assessment [18].

Nine studies performed analysis from surveillance systems data of the United States which differed in the time periods covered and the age group of the women included. Eight studies used national Breast Cancer Surveillance Consortium (BCSC) mammography registries which were linked to the Surveillance, Epidemiology, and End Results (SEER) pathology registries [8, 30, 32, 34, 37, 38, 40]. One study used the Vermont Breast Cancer Surveillance System (VBCSS) from the state of Vermont [33]. The studies included two types of analysis: first a case series of invasive BC that were used to evaluate the association between screening intervals and adverse tumour characteristics, and secondly, they estimated the 10-year cumulative probabilities of false positive results and false positive biopsy recommendations (Table 1) [43].

**Table 1:** Characteristics of the clinical trials, and observational studies identified in the literature search.

Author, year	Country, period	Design/screening intervals	No enrolled	Inclusion/exclusion criteria	Age ranges, (years)	Outcomes of interest
*Randomized clinical trial*						
BSFTG, 2002 [19], Duffy, 2008 [18]	United Kingdom 1989–2006	Attender women to prevalent screening at NHS breast screening program invited to conventional (3 years) interval or to three annual screenings.	-Annual: 37,530. -Triennial: 38,492.	I: women attending prevalent screening. E: women with BC diagnosed prior to the trial.	50–62	-BC mortality. -Interval cancer.
*Observational studies*						
Braithwaite, 2012	United States 1999–2006	Data from five BCSC registries (those matched to Medicare claims) linked to SEER programs/ tumor registries. -BC-case series: women with incident DCIS or invasive BC; interval groups defined as 1 (9–18 months) or 2 (19–30 months) years, based on the two most recent mammograms prior diagnosis. -FP-cohort: all first and subsequent screening mammography from 1999 to 2006, without BC diagnosis after 1 year of last examination.	-Annual (BC cases):1227. -Biennial (BC cases): 453. -FP-Cohort: 137,949.	I: women with at least two mammograms.	66–89	Reported by co-morbidity score categories: -Stage of BC (IIB+). -FP results. -FP recommendations.
Coldman, 2008 [31]	Canada	Pre-post screening policy changing evaluation. Data from the SMPBC linked to VSA registries over two time periods. From 1988 to 1997 women 50–79 years recommended annual screening, 1998–2005 changed to a biennial recommendation.	-Annual (before july 1996): 152,226 -Biennial (July 1996 or after): 184,764	E: women with a prior BC diagnosis not eligible to attend SMPBC.	50–79	-BC mortality. -Interval cancers.
Dittus, 2013 [32]	United States 1994–2008	Data from seven BCSC registries linked to SEER programs/tumor registries: -BC-case series: women with incident BC; intervals groups defined as 1 (9–18 months) or 2 (19–30 months) years based on the time between two most recent mammograms prior diagnosis. -FP-cohort: all screening mammography from 1994 to 2008, without BC diagnosis after 1 year of last examination.	-Annual (BC cases): 2766. -Biennial (BC cases): 1666. -FP-Cohort: 555,343	I: women with at least two mammograms before BC diagnosis. E: history of BC diagnosis, reporting hormone therapy use.	40–74	Reported by BMI categories: -Stage of BC (IIB+). -FP results. -FP recommendations.
Goel, 2007 [33]	United States 1994–2002	Data from the VBCSS which collects information from patients, radiologists and hospital pathology. -BC case series: women with incident BC, interval groups defined as 1 (0.75–1.49 years), 2 (1.5–2.49 years) years based on the time between two most recent mammograms prior diagnosis.	-Annual (BC cases): 1236 -Biennial (BC cases): 439	I: women with at least two mammograms before BC diagnosis. E: intervals of less than 273 days between mammograms. History of BC diagnosis.	>40 years	
Hubbard, 2011 [34]	United States, 1994–2006	Data from seven BCSC registries linked to SEER programs/tumor registries. -BC case series: women with incident invasive BC; interval groups defined as 1 (9–18 months) or 2 (19–30 months) years based on the time between two most recent mammograms prior diagnosis. -FP-cohort: screening mammograms from 1994 to 2004 or 2007 (depending on the registry).	-Annual (BC cases): 36,445 -Biennial (BC cases): 27,775 -FP-Cohort: 169,456	I: women with at least two mammograms. E: women with BC at or after 60 years.	40–59	-BC stage (IIB+). -FP results. -Bx recommendations.
Hunt, 1999 [35]	United States 1985–1997	Retrospective analysis from prospectively collected data of women that choose annual or biennial screening mammography, performed by University of California San Francisco Medical Center at screening mammography mobile van.	-Annual: 19,905 -Biennial: 4306	I: previous normal screening mammography, asymptomatic physician referred women from six contiguous counties. E: –	40–79	-Interval cancers
Kerlikowske, 2013 [8]	United States 1994–2008	Data from BCSC registries linked to SEER programs/tumor registries. -BC case series: women with incident DCIS or invasive BC; interval groups defined as 1 (0.75–1.49 years) or 2 (1.5–2.49 years) years based on the time between two most recent mammograms prior diagnosis. -FP-cohort: all first and subsequent screening mammography from 1994 to 2008, without BC diagnosis after 1 year of last examination.	-Annual (BC cases): 7039 -Biennial (BC cases): 3476 -Triennial (BC cases): 959 -FP-Cohort: 922,624	I: women with diagnosis of incident invasive or DCIS BC and at least 2 prior mammograms. E: History of BC diagnosis.	40–74	Reported by breast density categories: -BC stage(IIB+). -FP results. -Bx recommendations.
Parvinen, 2011 [39] Klemi, 1997 [36]	Finland 1987–2003	Population based, quasi-experimental. Mailed screening invitation to women aged 50 or more at biennial interval. Women less than 50 years, and born even-numbered year invited to annual screening and those born odd-numbered year were invited to triennial screening.	-Triennial: 6,926 -Annual: 7839	I: – E: –	40–49	-BC mortality. -Interval cancer.
McGuinnes, 2018 [42]	United States 2014–2015	Retrospective cohort. Women were approached during routine screening mammography at Columbia University Medical Center in New York. Annual interval from 9 to 18 months, biennial from 18 to 30 months. More than 913 days (3+ years) were considered non-compliant. Less than 274 days was considered recall imaging.	-Annual: 1399 -Biennial: 335	I: no previous diagnosis of BC, age ≥18 years	<50 years ≥50 years	-FP results.
Miglioretti, 2015 [37]	United States 1996–2012	Data from seven BCSC registries linked to SEER programs/tumor registries. -BC-case series: women with incident DCIS or invasive BC; intervals groups defined as 1 (11–14 months) or 2 (23–26 months) years based on the time between two most recent mammograms prior diagnosis.	-Annual (BC cases): 12,070 -Biennial (BC cases): 3370	I: women with at least two mammograms.	40–85	Reported by menopausal and HT use categories: -BC stage(IIB+). -Interval cancer.
O’Meara, 2013 [38]	Unitesd States 1994–2008	Data from seven BCSC registries linked to SEER programs/tumor registries. -BC-case series: women with incident DCIS or invasive BC; interval groups defined as 1 (9–18 months) or 2 (>18–30 months), or 3 (>30–42 months) years based on the time between two most recent mammograms prior diagnosis. -FP-cohort: all screening mammography from 1994 to 2008, without BC diagnosis after 1 year of last examination.	-Annual (BC cases): 8876 -Biennial (BC cases): 4265 -Triennial (BC cases): 1255 -FP-cohort: 1,276,312	I: women with at least two mammograms.	40–74	Reported by breast density and ethnic categories: -BC stage(IIB+). -Interval cancer. -FP results. -Bx recommendations.
Sanderson, 2015 [41]	Unites States 1995–2000	Data from Medicare claims and SEER. -BC-case series: women with incident BC; groups defined based on mammography screening periodicity over the 4 years prior to diagnosis as (a) no or irregular mammography screening, (b) biennial mammography, and (c) annual mammography.	-Irregular (BC cases): 29,712 -Biennial (BC cases): 11,227 -Annual (BC cases): 23,355.	I: non-Hispanic white or black ethnicity, complete Medicare coverage during 4-year before BC diagnosis; primary BC diagnosed between 69 to 84 years. E: BC diagnosed by autopsy or death certificate, stage IV cancer.	69–84	Reported by ethnicity category (non-Hispanic white or black women): -Mortality among BC cases (according to screening interval history).
White, 2004 [40]	Unites States 1996–2001	Data from seven BCSC registries linked to SEER programs/tumor registries. -BC series: women with incident DCIS or invasive BC; intervals groups defined as 1 (9–18 months) or 2 (>18–30 months) years based on the time between two most recent mammograms prior diagnosis.	-Annual (BC cases): 5400. -Biennial (BC cases): 2440.	I: women with diagnosis of invasive or DCIS BC and at least two prior mammograms. E: history of BC.	40–89	Reported by breast density: -BC stage(IIB+). -Interval cancer.

*SMPBC* Screening Mammography Program of British Columbia, *VSA* Vital Statistics Agency, *SEER* Surveillance Epidemiology and End Results, *BCSC* Breast Cancer Surveillance Consortium, *VBCSS* Vermont Breast Cancer Surveillance System, *DCIS* ductal carcinoma in situ; *BC* breast cancer, *NHS* National Health Services, *FP* false positive, *Bx* biopsy, *I* inclusion criteria, *E* exclusion criteria.

A quasi-experimental study included women aged 40–49 who were invited to attend a screening programme in Finland. Those women born in an even calendar year were invited for mammography screening every year, while those born in an odd calendar year were invited to screening every 3 years [39]. One study conducted a comparative analysis of two time periods in British Columbia-Canada, before and after 1997, year when the Screening Mammography Program of British Columbia (SMPBC) changed its policy from annual to biennial mammography for women aged 50–79 [31].

Two studies included women from screening programmes at medical centers from the US. The first performed a retrospective analysis of data from women who chose to attend either annual or biennial mammography examinations in a screening programme of the University of California San Francisco Medical Center [35]. The second study was a retrospective cohort of women without previous diagnosis of BC who attended a routine screening examination at Columbia University Medical Center in New York; the screening interval was defined using the time elapsed since their previous exam according to their electronic clinical records [42] (Table 1).

Six studies used microsimulation models developed within the Cancer Intervention and Surveillance Modelling Network (CISNET) collaboration: Model D (Dana-Farber) [44], Model E (Erasmus) [45], Model GE (Georgetown-Einstein) [46], Model M (MD Anderson) [47], Model S (Stanford) [48], and Model W (Wisconsin-Harvard) [49]. Each of these models has its own characteristics which are described elsewhere [50], they vary in the model structures and assumptions such as factors conditioning screen detection, individual risk factors or allowing spontaneous regression of ductal carcinoma in-situ (DCIS) lesions [51]. Four studies assessed mammography screening intervals for the U.S. population reporting the median estimates from two to six models [7, 21, 22, 24]. Two studies simulated screening for a Canadian population based on an adaptation of Model W [26, 28]. One microsimulation study projected adverse events related to radiation exposure from mammography exams in women 50–74 years of age (Table 2) [21]. One additional study adapted a microsimulation Markov model to the German context to assess annual, biennial, and triennial routine screening in women aged 50–69 [29].

**Table 2:** Characteristics of decision analysis studies identified.

Author, year	Modelled population	Design/screening intervals	Strategies	Parameters	Years of screening	Outcomes of interest
*Non-individual based models*						
Gunsoy, 2014 [20]	United Kingdom	Markov-cohort simulation model. -Healthy, preclinical non-progressive in situ, preclinical progressive in situ, preclinical invasive, diagnosed in situ, and diagnosed invasive breast cancer by NPI category, death from BC, and death from other causes.	Six strategies defined by: -Starting age: 40, 47, 50 years. -Interval: triennial, annual, and hybrid (annual for 40–47, triennial thereafter) -Screening scenarios stopped at 70 or 73 years.	-(F): up to 85 years old -(SA): on uptake, sensitivity, and sojourn time. -(O): difference in the cumulative incidence of invasive in situ cancer. -(MA): NHS breast cancer program.	40–73	-Breast cancer deaths averted -Overdiagnosis-
Tsunematsu, 2015 [23]	Japan, United States.	Transition cohort model to simulate impact of screening for the Japanese and US population. The source of stage distributions were data from the Japanese Breast Cancer Society and the BCSC and National Cancer Data BASE respectively.	Twelve strategies defined by: -Starting age: 40, 50 years -Intervals: annual, biennial -Screening stopping at 69, 74, and 79 years.	-(F): NR -(O): NR -(SA:): mortality rate of undetected BC -(MA): SE: 81.5%; SP 90.4–94.7% (Japan)	40–79	-Breast cancer deaths averted -FP results
Vilaprinyo, 2014 [27]*	Spain	Stochastic transition model. Extension of the Lee and Zelen model to estimate incidence and prevalence,	Twenty strategies defined by: -Starting ages: 40, 45, 50. -Intervals: annual, biennial -Screening stopping at 69, 70, 74 and 79.	-(C): born from 1948 to 1952 -(F): time horizon was 40–79 years -(O): NR (SA): not provided by authors -(MA): SE: 0.55 for 40–45 years, 0.70 for 45–50 years, 0.75 for 50–70 years and 0.80 for >70 year -(Q): includes anxiety, and FP results	40–79	-Breast cancer deaths averted -Overdiagnosis -QALYS -FP results -Benign breast bx
Yaffe, 2011 [25]	Canada	Model by Preston (excess absolute risk of radiation induced BC). Applied to Canadian population of 2002. Digital mammography.	Six strategies defined by: -Starting ages: 40, 50 -Intervals: annual, biennial (hybrid annually in 40 s, biennial thereafter). -Screening stopping at 49, 59 years.	-(F): screening began up to 109 years -(SA): using relative model instead of absolute model, latency years, survival rates.	40–74	- Radiation induced BC - Radiation induced BC deaths
**Individual based models**						
Arnold, 2019 [29]	Germany *N* = 3,000,000 women	A microsimulation-Markov model included 6 health states: healthy (no breast cancer); ductal carcinoma in situ (DCIS); localized, regional, or distant invasive breast cancer; and death.	Three regular screening strategies and additional strategies based on individual risk assessment (not shown) -Interval: annual, biennial, triennial -Starting age: 50 years -Screening stopping: 69 years	-(F): from age of 50, until the end of life or 100 years. -(T): specific treatment based on hormone receptors -(MA): digital mammography sensitivity based in BCSC. -(O): -(SA): univariate and probabilistic sensitivity analysis (e.g. DCIS incidence, invasive cancer incidence, invasive cancer morality)		-QALY -Biopsy after false positive screening
Mandelblatt, 2016 [7]	United States *N* = 1000 women	Six micro simulation models developed within the CISNET collaboration: model D, model E, model GE, model M, model S and model W. Updating of models include 1) portrayal of molecular subtypes based on ER and HER2 status, current population incidence, digital screening, and update therapies.	Eight strategies defined by: -Starting age: 40, 45, or 50 years -Interval: annual, biennial and hybrid (annual in 40 s, biennial thereafter). -All strategies stop screening at 74.	-(C): born in 1970 of average-risk and average breast density. -(F): from age 25 years until death or age 100 -(O): models assume proportions of DCIS non-progressive; models M and W assumed some non-progressive invasive cancers -(T): specific treatment based on hormone receptors -(MA): digital mammography sensitivity based in BCSC. -(ME): RR 0.72 (95%CI 0.65–0.75)	40–74	Reported as median across models: -BC deaths averted -Overdiagnosis -QALYS (includes overdiagnosis) -FP results -Benign breast bx
Miglioretti, 2016 [21]	United States *N* = 100,000 women	Two micro simulation modeling approaches for digital mammography. MISCAN-Fadia model and a new model for radiation exposure (which accounts for repeated mammography or radiation exposure and BS). Excess of radiation induced BC using the results from Preston.	Eight strategies defined by: -Starting age: 40, 45, or 50 years -Interval: annual, biennial and hybrid (annual in 40 s, biennial thereafter). -All strategies stop screening at 74.	-(MA): digital mammography sensitivity based in BCSC -(BS): views and compressed thickness from DMIST -(RD): product of half the number of views of both breast by dose per view.	40–74	- BC deaths averted - Radiation induced BC - Radiation induced BC deaths
Mittmann, 2018 [28]**	Canada *N* = 2,000,000 women	One modified microsimulation, from the perspective of the Ontario public health care system model W developed within the CISNET collaboration. Discrete event, stochastic simulation based on the US population. Model simulated the lives of women at 6-month intervals.	Eleven screening scenarios: -Annual, biennial, triennial, and hybrid of these -Starting age: 40 or 50 years. -Screening stopping at: 49, 69 or 74 years.	-(T): specific treatment based on hormone receptors -(C): all women born in 1960 validated against US data and modified against Canadian data. -(F): lifetime horizon -(SA): input cost varied for key resources in one-way analysis -(MA): digital mammography sensitivity based in BCSC.	40–74	-QALYs
Trentham-Dietz, 2016 [22]	United States *N* = 1000	Three micro simulation models developed within the CISNET collaboration: model GE, model W, model E. Model applied to population subgroups based on 4 breast density levels and 4 exemplar relative risk levels: average, postmenopausal obesity, history of benign breast biopsy result, history of lobular carcinoma in situ.	Six screening scenarios: -Annual, biennial, or triennial digital mammography. -Starting age: 50 or 65 (received biennial from 50 to 64). -Stopping age: 74.	-(C): born in 1970 -(F): from age 25 years until death or age 100 -(O): models assume proportions of DCIS non-progressive; models W assumed some non-progressive invasive cancers -(T): specific treatment based in hormone receptors -(MA): digital mammography accuracy based in BCSC.	40–74	Reported as median across models (stratified by BD): -BC deaths averted. -Overdiagnosis. -QALYS (includes overdiagnosis). -FP results. -Benign breast bx.
Van Ravestein, 2012 [24]	United States *N* = 1000 women	Four micro simulation models developed within the CISNET collaboration: model D, model E, model GE and model W. The models include biennial screening for women 50 to 74 years extended with 4 screening scenarios varying by screening interval (annual and biennial) and screening method (film and digital).	Five screening scenarios: -Interval: annual and biennial -Film or digital mammography -Age group: 40–49 years All scenarios estimated incremental effects compared to 50–74 screening.	-(T): specific treatment based in hormone receptors -(C): born in 1960 of average-risk. -(MA): digital and film mammography accuracy based in BCSC.	40–49	-BC deaths averted. -FP results.
Yaffe, 2015 [26]	Canadian, *N* = 2,000,000 women	One model from the CISNET collaboration (model W), adapted to the Canadian context. Treatment effectiveness was implemented on a cure/no cure model. The model allowed different proportion of hormone receptors subgroups.	Eleven screening scenarios: -Interval: annual, biennial, triennial (and two hybrid scenarios). -starting age: 40 or 50 years. -stopping age: 69 or 74 years.	-(F): from age 40 years until death or age 99. -(C): born in 1960 of average-risk.	40–74	-BC deaths averted. -FP results.

Model D: Dana-Farber Cancer Institute Boston Massachusetts; Model E: Erasmus Miscan-Fadia; University Medical Center Rotterdam, the Netherlands; Model GE: Georgetown University Medical Centre, Washington, DC, and Albert Einstein College of Medicine, Bronx, New York; Model M: MD Anderson Cancer Center, Houston, Texas; Model W: University of Wisconsin; CISNET: Cancer Intervention and Surveillance Modeling Network; BCSC: Breast Cancer Surveillance Consortium; ER: oestrogen receptor; HER2: human epidermal growth factor receptor 2; NPI: Nottingham prognostic index; QALY: quality adjusted life-years; (SA): sensitivity analysis; (C): women born cohort; (F): time of follow-up, horizon time; (MA): mammography accuracy; (ME): mammography effectiveness; (O): overdiagnosis assumptions; (T): tailored treatment; (RD): radiation dose; BC: breast cancer; FP: false positive.

*Unpublished data were provided by the authors.

**A previous study by the same authors and using the same model and population was excluded (Mittmann 2015) as the updated study provided a more detailed description of the outcomes.

The remaining four modelling studies implemented non-individual models. One transition model evaluated annual versus biennial screening intervals in Japan [23]. One Markov model assessed breast cancer deaths averted and overdiagnosis due to screening for women in the United Kingdom [20], and another study applied the model developed by Preston to estimate radiation related events [25]. We obtained non-publicly available data of a transition modelling study for a Spanish cohort described elsewhere (Table 2) [27, 52].

### Benefits and harms in women aged 45–49 (Tables 3/4)

#### Observational studies

A Finish study suggested an increase in the risk of BC mortality in annual versus triennial screening (incidence RR 1.14; 95%CI 0.59–2.19) although the estimate was very uncertain [39]. The odds of advanced breast cancer stage (IIB–IV) may be higher in women with a history of biennial screening compared to annual screening (OR 1.17; 95%CI 0.93–1.46) among incident breast cancers from US registries [37].

**Table 3:** Summary of the desirable and undesirable effects from RCTs and observational studies for different screening intervals and age groups.*

Age group	Annual vs. Biennial			Triennial vs. Biennial				Annual vs. Triennial			Certainty of evidence
	N° of studies, countries	Relative effect (95%CI)	Absolute reduction (95%CI)	N° of studies, countries	Relative effect (95%CI)		Absolute reduction (95%CI)	N° of studies, countries	Relative effect (95%CI)	Absolute reduction (95%CI)	
*Breast cancer (BC) mortality*											
40–49	–	–	–	–	–		–	1 (Finland) [39]	RR 1.14 (0.59 to 2.19)**	3 more (7 fewer to 21 more)	Very low
50–69	1 (Canada) [31]	IRR: 0.94 (0.68 to 1.31)	4 fewer (22 fewer to 21 more)	–	–		–	1 (UK) [18]***	RR 0.93 (0.76 to 1.12)	42 fewer (144 fewer to 72 more)	Very low for annual vs biennial and moderate vs triennial
*BC stage (IIB–IV)*											
40–49	1 (US) [37]	OR 0.85 (0.75 to 0.96)	NE	1 (US) [38]	OR 0.78 (0.54 to 1.11)		NE	–	–	–	Very low for all comparisons
50–69	1 (US) [37]	OR: 0.93 (0.81 to 1.09)	NE	1 (US) [38]	OR: 0.83 (0.65 to 1.07)		NE	–	–	–	Very low for all comparisons
70–74	1 (US) [37]	OR 0.98 (0.76 to 1.27)	NE	–	–		–	–	–	–	Very low
*Interval cancer*											
40–49	1 (US) [35]	RR 0.46 (0.16 to 1.36)	81 fewer (126 fewer to 54 more)	–	–	–		–	–	–	Very low
50–69	1 (US) [37]	A: 22% (21% to 30%) of BC cases B: 27% (26% to 29%) of BC cases		1 (US) [7]	T: 44% (41% to 48%) of BC cases B: 41% (39% to 42%) of BC cases			1 (US) [7]	A: 30% (29% to 31%) of BC cases T: 44% (41% to 48%) of BC cases		Very low for all comparisons
70–74	1 (US) [30]	A: 23% (22% to 25%) B: 33% (30% to 36%)		–	–	–		–	–	–	Very low
*False positive results*—*10 year cumulative probability per woman*											
40–49	1 (US) [32]	A: 67% (65% to 68%) B: 45% (44% to 46%)		1 (US) [32]	T: 30% (29% to 30%) B: 41% (41% to 42%)			1 (US) [38]	A: 65% (63% to 65%) T: 29% (29% to 30%)		Very low for all comparisons
50–69	1 (US) [32]	A: 54% (53% to 55%) B: 34% (34% to 35%)		1 (US) [38]	T: 25% (25% to 25%) B: 35% (35% to 36%)			1 (US) [38]	A: 55% (55% to 56%) T: 25% (25% to 25%)		Very low for all comparisons
70–74	1 (US) [30]	A: 47% (45% to 50%) B: 27% (26% to 28%)		–	–	–		–	–	–	Very low
*False positive biopsy recommendation*—*10 year cumulative probability per woman*											
40–49	1 (US) [32]	A: 11% (10% to 13%) B: 6% (5% to 7%)		1 (US) [32]	T: 4% (4% to 4%) B: 6% (6% to 6%)			1 (US) [38]	A: 11% (11% to 12%) T: 4% (4% to 4%)		Very low for all comparisons
50–69	1 (US) [32]	A: 8% (7% to 9%) B. 5% (4% to 5%)		1 (US) [38]	T: 4% (4% to 4%) B: 5% (5% to 6%)			1 (US) [38]	A: 10% (9% to 10%) T: 4% (4% to 4%)		Very low for all comparisons
70–74	1 (US) [30]	A: 9% (8% to 11%) B: 4% (4% to 5%)		–	–	–		–	–	–	Very low

To review the reference for each study and the reasons for downgrading the certainty of the evidence see Supplementary file Table S4–S12.

*Only the study with the longest time of observation was included when there were several publications with overlapping time periods. When studies provided results stratified by women´s characteristics, we extracted data from subgroups more similar to European context (i.e. white women instead of other ethnic groups).

**We calculated the confidence interval from the raw data reported in the publication as the original interval was not consistent with the main effect and lower interval bound.

***Randomized clinical trial study.

**Table 4:** Summary of desirable and undesirable effects (number of events) from modelling studies for different screening intervals and age groups (per 100,000 screened women).**

Age group	Annual vs. Biennial					Triennial vs. Biennial				Annual vs. Triennial			Certainty of evidence across comparisons
	N° of studies, countries		N° of events per arm		Absolute reduction	N° of studies, countries	N° of events per arm		Absolute reduction	N° of studies, countries	N° of events per arm	Absolute reduction	
*Breast cancer deaths averted (BC)*													
45–49*	2 (US) [7, 21]^##^		A: 70–90 B: 39–40		30 more to 51 more	1 (Spain) [27]	T: 47 B: 52		5 fewer	1 (Spain) [27]^#^	A: 33 T: 47	14 fewer	Very low for all comparisons
50–69	3 (Canada, Japan, Spain) [23, 26, 27]		A: 631–870 B: 426–705		165 more to 205 more	2 (Canada, Spain) [26, 27]	T: 397–400 B: 426–520		120 fewer to 29 fewer	2 (Canada, Spain) [26, 27]	A: 631–740 T: 397–400	234 more to 340 more	Very low for all comparisons
70–74*	2 (Canada, Spain) [26, 27]		A: 100–142 B: 90–145		3 fewer to 10 more	2 (Canada, Spain) [26, 27]	T: 80–136 B: 90–145		10 fewer to 9 fewer	2 (Canada, Spain) [26, 27]	A: 100–142 T: 80–136	6 more to 20 more	Very low for all comparisons
*Overdiagnosis*													
45–49*	2 (Spain, US) [7, 27]		A: 143–200 B: 0–119		24 more to 200 more	1 (Spain) [27]	T: 88 B: 119		31 fewer	1 (Spain) [27]	A: 143 T: 88	55 more	Very low for all comparisons
50–69	1 (Spain) [27]		A: 904 B: 609		295 more	1 (Spain) [27]	T: 500 B: 609		109 fewer	1 (Spain) [27]	A: 904 T: 500	404 more	Very low for all comparisons
70–74*	1 (Spain) [27]		A: 269 B: 236		33 more	1 (Spain) [27]	T: 193 B: 236		43 fewer	1 (Spain) [27]	A: 269 T: 193	76 more	Very low for all comparisons
*QALYs*													
45–49*	2 (Spain, US) [7, 27]		A: 727–1540 B: 665–1060		62 more to 480 more	1 (Spain) [27]	T: 653 B: 665		12 fewer	1 (Spain) [27]	A: 727 T: 653	74 more	Very low for all comparisons
50–69	3 (Canada, Germany, Spain) [27–29]		A: 4400–7100 B: 3900–5000		500 to 2100 more	3 (Canada, Germany, Spain) [27–29]	T: 3300–4386 B: 3900–5000		1200–328 fewer	3 (Canada, Germany, Spain) [27–29]	A: 4,400–7,100 T: 3,300–4,386	1,100 to 3,100 more	Very low for all comparisons
70–74*	2 (Canada, Spain) [26, 27]		A: 336–600 B: 427–500		91 fewer to 100 more	2 (Canada, Spain) [26, 27]	T: 300–398 B: 427–500		200 fewer to 29 fewer	2 (Canada, Spain) [26, 27]	A: 336–600 T: 300–398	62 fewer to 300 more	Very low for all comparisons
*False positive results*													
45–49*	2 (Spain, US) [7, 27]		A: 9150–56,700 B:6301–26,700		2849 more to 30,000 more	1 (Spain) [27]	T: 4831 B: 6301		1470 fewer	1 (Spain) [27]	A: 9,150 T: 4,831	4,319 more	Very low for all comparisons
50–69	3 (Canada, Japan, Spain) [23, 27, 29]		A: 42,606–152,800 B: 29,039–89,500		13,567 more to 63,300 more	2 (Canada, Spain) [26, 27]	T: 24,252–69,900 B: 29,039–89,500		19,600 fewer to 4787 fewer	2 (Canada, Spain) [26, 27]	A: 42,606–152,800 T: 24,252–69,900	18,354 to 82,900 more	Very low for all comparisons
70–74*	2 (Canada, Spain) [26, 27]		A: 5766–24,500 B: 3459–17,400		2307 more to 7100 more	2 (Canada, Spain) [26, 27]	T: 2295–12,700 B: 3459–17,400		4700 fewer to 1164 fewer	2 (Canada, Spain) [26, 27]	A: 5,766–24,500 T: 2,295–12,700	3,471 more to 11,800 more	Very low for all comparisons
*Benign biopsy recommendations*													
45–49*	2 (Spain, US) [7, 27]		A:409–5600 B: 208–3000		201 more to 2600 more	1 (Spain) [27]	T: 108 B: 208	100 fewer		1 (Spain) [27]	A: 409 T: 108	301 more	Very low for all comparisons
50–69	3 (Canada, Germany, Spain) [26, 27, 29]		A: 904–16,300 B: 609–14,400		295 more to 4900 more	3 (Canada, Germany, Spain) [26, 27, 29]	T: 2166–14,100 B: 2487–14,400	1300 fewer to 300 fewer		3 (Canada, Germany, Spain) [26, 27, 29]	A: 3,455–16,300 T: 2,166–14,100	1,289 fewer to 5,900 fewer	Very low for all comparisons
70–74*	2 (Canada, Spain) [26, 27]		A: 428–3200 B: 287–3500		No difference to 142 more	2 (Canada, Spain) [26, 27]	T: 171–3200 B: 287–3500	300 fewer to 116 fewer		2 (Canada, Spain) [26, 27]	A: 428–3,200 T: 171–3,200	0 more to 257 more	Very low for all comparisons
*Radiation induced BC*													
45–49*	1(US) [21]		A: 32 B: 18		14 more	–	–	–		–	–	–	Very low for all comparisons
50–69	2 (Canada, US) [21, 25]^‡^		A: 27–49 B: 14–27		13 more to 22 more	–	–	–		–	–	–	Very low for all comparisons
*Death by radiation induced BC*													
45–49*	1 (US) [21]		A: 6 B: 4		2 more	–	–	–		–	–	–	Very low for all comparisons
50–69	2 (Canada, US) [21, 25]^‡^		A: 3–7 B: 2–4		1 more to 3 more	–	–	–		–	–	–	Very low for all comparisons

To review the reference for each study and the reasons the certainty of the evidence was downgraded see: Supplementary file Table S4 to S12. When more than one study informing an outcome, the number represents the range of point estimates reported across studies.

*Number of events was not directly reported for this age group. We made an ad-hoc calculation subtracting the events from overlapping age groups (e.g. number of QALYS in women 45 to 69 years minus the estimates from 50 to 69 years).

**The certainty of evidence departed from *low* as the input parameters that inform the modelling studies were of low to very low certainty.

# Only one study providing unpublished data informed this comparison. The result was in a different direction than the other bodies of evidence and thus cautious interpretation is recommended.

^##^Unpublished data from one study (Vilaprinyo 2014) reported 19 fewer BC deaths averted with annual compared to biennial screening. This result was inconsistent with the other studies and, therefore, is not included in the table.

In women of normal weight, the 10-year probability of false positive results was 11.2% (95%CI 9.8–12.8%) with annual screening and 6.0% (95%CI 5.4–6.6%) with biennial screening [32]. The probability of a false positive biopsy recommendation was 11.4% (95%CI 10.5–12.4%) with annual screening, 5.9% (95%CI 5.6–6.2%) with biennial screening, and 3.9% (95%CI 3.7–4.1%) with triennial screening among white women [38].

Moreover, indirect evidence from the wider age group of women (40–79) suggested that the incidence of interval cancers may be lower among annually screened (0.07%) compared to biennially screened (0.15%) women, but it was very uncertain given the small number of events [35].

#### Modelling studies

One study estimated, across six microsimulation models, a median of 30 more deaths averted per 100,000 women undergoing annual screening compared to biennial screening in the US population [7], while the median number of additional QALYs gained with annual screening was 480 more compared to biennial screening [7]. In the same modelling study, the overdiagnosis estimation was higher with annual screening compared to biennial screening [7]. One modelling study assessed the risk of radiation induced adverse events in this age group and found that annual screening yielded 14 more induced BC and 2 more deaths per 100,000 screened women compared to biennial screening [21].

### Benefits and harms in women aged 50–69 (Tables 3/4)

#### Randomised clinical trials

Duffy et al. reported in the UKCCR study, over a median of 162 months of follow-up, that annual screening may decrease the risk of BC mortality compared to triennial screening among attenders to the prevalent screening (RR = 0.89, 95% CI 0.73−1.07) [18]. Moreover, there was a small difference in the size of the tumour at diagnosis, with a major proportion of them being 10 mm or smaller in the annual screening group compared to the triennial group (25% vs. 19%) [18, 19].

#### Observational studies

One study in a province of Canada comparing the period before and after mammography screening changed from annual to biennial found there may be little to no difference in mortality (MR 1.06; 95%CI 0.76, 1.46) or interval cancer (RR 0.98; 95%CI 0.90–1.06) between the two-time periods [31].

Miglioretti et al. found there may be no difference in the risk of advanced BC stage (IIB–IV) in the age groups 50–69 (adjusted RR 0.98; 95%CI 0.80–1.21) and 60–69 (adjusted RR 0.99; 95%CI 0.79–1.24) with annual versus biennial screening [37]. Another study in the US found uncertain evidence that triennial screening compared to biennial screening in white women might be associated to lower odds of stage IIB-IV (OR 0.83; 95%CI 0.65–1.07) but it was not consistent with the observed difference in large tumour size (>20 mm) (OR 1.15; 95%CI 0.93–1.41), or presence of lymph nodes (OR 0.98; 95%CI 0.80–1.21) at BC diagnosis [38].

From a US study using mammography and tumour registries, the 10-year probability of a false positive result was 55.2% (95%CI 54.8–55.7%) with annual screening, 35.4% (95%CI 35.0–35.7%) with biennial screening, and 24.8% (95%CI 24.5–25.2%) with triennial screening [38]. The cumulative 10-year probability of having a false positive biopsy recommendation was 9.7% (95%CI 9.3–10.1%) with annual screening, 5.4% (95%CI 5.2–5.6%) with biennial screening, and 3.7% (95%CI 3.6–3.9%) with triennial screening [38]. These findings were consistent with the risk of false positive results observed in a retrospective cohort of a screening programme of New York [42].

#### Modelling studies

In a Canadian modelling study, the number of BC deaths averted per 100,000 women aged 50–69 screened annually, biennially or triennially compared to no screening was 740, 520 and 400, respectively [26]. In another study, including three models tailored to the US population, the number of BC deaths averted per 100,000 screened women aged 50–74, with scattered fibroglandular breast density, was 690, 520 and 400 for annual, biennial and triennial screening [22] and the number of QALYs gained was 6000, 4700 and 3600, respectively [22]. A microsimulation model for the German population found a median of 4400, 3900 and 3330 additional QALYs with annual, biennial and triennial screening [29].

The estimated overdiagnosis was greater with more frequent screening intervals. In women with scattered fibroglandular density aged 50– 74, a microsimulation model study estimated 2900, 2000 and 1600 for annual, biennial and triennial screening compared to no screening per 100,000 women [22]. A similar trend was reported in a study using non-individual models for a Spanish cohort of women aged 50–69 [27].

A microsimulation model estimated the risk of radiation induced adverse events in 100,000 women aged 50–74 to be of 27 induced BC cases with biennial screening and 49 with annual screening [21]. The attributed number of radiation related deaths simulated was 4 with biennial screening and 7 with annual screening for the same age group [21]. A similar difference between biennial and annual screening intervals was observed from an excess absolute risk model of radiation induced BC [25].

### Benefits and harms in women aged 70–74 (Tables 3/4)

#### Observational studies

Three studies provided estimations of advanced BC stage (IIB–IV) in older women, using population registries but for different age ranges (i.e. 66–89 [30], 70–85 [37] and 70–89 years [40]). In the age group of 70–85, the proportion of tumours at stage IIB–IV were no different among newly diagnosed BC with a history of biennial or annual screening (OR 0.98 95%CI 0.76–1.27) [37].

One study estimated that the 10-year cumulative probability of false positive results for women between the ages of 75 and 89 may be higher with annual screening (47%, 95%CI 44.9–49.5%) compared to biennial screening (26.6%, 95%CI 25.7–27.5%) [30]. The cumulative probability of false positive biopsy recommendations may also be higher for annual screening (9%, 95%CI 8–11%) compared to biennial screening (4%, 95%CI 4–5%) [30].

#### Modelling studies

The estimated difference for BC deaths between the different intervals might be small. A microsimulation model estimated the number of BC deaths averted for annual, biennial and triennial screening to be 100, 90 and 80, respectively, compared to no screening per 100,000 screened Canadian women [26]. This result was consistent with the one reported in a non-individual model for a Spanish cohort which showed almost similar benefits for the three screening intervals (unpublished data) [27], and a small number of QALYs gained since life expectancy is lower in this age group.

Only one non-individual based model estimated overdiagnosis for this age group and it showed a small increasing trend with shorter screening intervals from 193 for triennial screening to 269 for annual screening [52].

### Risk of bias and certainty of the evidence

Overall, the certainty of the evidence was very low, and therefore the differences observed between the possible combinations of screening intervals and age groups are uncertain. The exemption was the evidence from the only RCT included in this systematic review which was downgraded to moderate certainty due to imprecision [19].

The evidence from observational studies was limited among other factors by indirectness as for the age group of 45–49 we only identified studies including a broader age range from 40 to 49 years of age at the time of invitation to screening, and from some studies we had to extract results from specific subgroups of women (e.g. normal weight or white women). All secondary analysis from surveillance registries were also subject to misclassification bias of the interventions as the periodicity of screening was assigned based on different time ranges that elapsed between the two latest mammographies prior to diagnosis. Additionally, US studies used opportunistic screening, thus women might have anticipated or delayed the mammography due to preferences or indications given by radiologists.

We decided that for modelling studies, our GRADE assessment departed from low certainty after considering methodological limitations of key input evidence (i.e. mammography sensitivity estimated from BCSC registries including women from wider age groups than our clinical question and with a clinical follow-up restricted to only one year [53], or no formal assessment of risk of bias in the individual-patient-data meta-analysis used to inform treatment effectiveness [54]) and that credibility assessment of model development was limited due to suboptimal reporting. There was also limited reporting of formal sensitivity analysis to assess the impact of input data assumptions on the simulated events [21, 24, 25]. We had concerns about indirectness given that most models used observational data from the US to inform their input parameters (i.e. radiation induced BC), and because in one modelling study data was only available by different levels of breast density (i.e. scattered fibroglandular density) [22]. Finally, one study providing unpublished data (Vilaprinyo 2014) [27] reported fewer BC deaths averted with annual compared to biennial or triennial screening in the age group of 45–49 years. This result was not internally consistent (i.e. annual screening had the largest number of BC deaths averted from 45 to 69) and differed from other studies or bodies of evidence; thus we included this result cautiously only if other studies were not available (Table 4).

The detailed risk of bias assessment per study is available under request. The evidence profiles for all age groups and intervals comparisons describing the reasons for downgrading the certainty of evidence are available from Supplementary Tables S5–S13. In the evidence profiles we prioritised the reporting of evidence from observational/randomised studies over modelling studies (i.e. false positive results).

## Discussion

### Main findings

Our systematic review shows that in women of average breast cancer risk, screening intervals may have different trade-offs between benefits and harms for each age group. However, the available evidence was mostly of very low certainty and precludes us from reaching firm conclusions. In women 50–69 years old, annual compared to biennial screening may have small additional benefits but an important increase in false positive results. Triennial compared to biennial screening suggests the latter provides more benefits but also some additional harms. In younger women (45–49), the more frequent screening intervals (going from biennial to annual screening) provides smaller incremental benefits (i.e. number of BC deaths averted), nearly similar incremental estimates of overdiagnosis and slightly more incremental harms (i.e. false positive results and false positive biopsies recommendations from observational studies) than in women 50–69 years of age. Thus the overall balance between benefits and harms is more favourable in the latter age group. Finally, among women aged 70–74, the smaller incremental harms and similar benefits with shorter screening intervals suggests that longer intervals probably have a more favourable overall balance, but the difference may be small.

We observed sparse data, especially in older women and for critical outcomes, such as BC mortality or disease stage at diagnosis. The only included RCT showed that annual screening, compared to triennial screening, probably reduces BC mortality in women 50–62 years of age. Observational evidence consisted of population registries from different time periods with high uncertainty. We considered modelling evidence when empirical evidence was not available. However, its certainty was very low due to indirectness, since data for input parameters mostly come from opportunistic screening settings,. Model studies suggested that in women aged 50–69 the benefits with annual screening may be a bit larger but may also be associated to relevant harms, including the possibility of a small increase of new BC lesions induced by radiation exposure; thus, biennial screening may provide a more favourable balance, while in other age groups the potential benefits gains with more frequent screening intervals may be smaller.

### Our results in the context of previous research

Our results are broadly consistent but more comprehensive than previous reviews. The USPFTF based their assessment on one modelling study (included in our review), concluding that when moving from biennial to annual mammography, regardless of the starting age, there is a small increase in averted deaths but with a large increase of harms [7]. A systematic review conducted by the American Cancer Society included an indirect comparison between RCTs and a model study from the CISNET collaboration, concluded that beginning screening with more frequent intervals likely results in a greater mortality reduction but the magnitude is uncertain [55].

The modelling estimates of harms due to overdiagnosis remains a matter of debate as there is no consensus on the methods to quantify this outcome [56], and many assumptions are made, including the clinical impact of DCIS and the probability of some cancers to spontaneously regress [50]. It is worth noting that there is also considerable uncertainty in the evidence coming from RCTs. For example, a review including only studies that did not invite women of the control group to screening at the end of the trial period, reported a relevant proportion of overdiagnosis [57]. However, the UK age trial showed that the cumulative incidence of invasive cancers was similar, if not higher, in women who underwent only one mammogram after the age of 50 compared to women who underwent annual mammography from 40 to 49, and then entered a triennial screening programme [58].

The cost-effectiveness of implementing different screening intervals has been studied in few microsimulation models. One study assessed the impact of extending the Dutch screening programme in women under 50, showing that biennial strategies were cost-effective while other alternatives, such as annual screening starting at 45, resulted in less favourable incremental cost-effectiveness ratios (ICERs) [59]. However, the study used an 80% adherence to screening [59], which might have influenced the relative trade-offs between different screening intervals, as previously described [22]. In women from the US between 50 and 74 years of age, with different breast densities and individual risk level of developing BC, triennial strategies were considered cost-effective (at a threshold of $100 000 per QALY) for subgroups with average risk and low breast density, while biennial strategies were cost-effective for other breast density subgroups at an average or intermediate risk [22].

### Limitations and strengths

Although we included only English language articles, the risk of selection bias is probably small as we also screened previous systematic reviews and consulted the GDG experts, not identifying additional studies. Some results are not directly transferable to the European context; for example the cumulative 10-year false positive rates from US studies are higher than those reported in organised European screening programmes. However, we assumed that the difference between intervals would be more comparable across different settings. The scarce available empirical evidence to evaluate the trade-offs between benefits and harms limited our conclusions. We therefore included modelling evidence to complement the gaps in the evidence, an approach that is recommended for interventions such as population screening [60].

### Implications for practice and research

Our findings may have different implications for practice depending on the age group, the balance between benefits and harms, available resources for public health services, and how women value the different outcomes. In the case of women invited to an opportunistic screening programme (or considering screening) a shared decision-making process to carefully explain the pros and cons of each decision is warranted. Similarly, given the low certainty of evidence and the variability and uncertainty of how women value outcomes at stake, guideline panellists are likely to formulate conditional recommendations, as opposed to strong ones. The scope of this review is determined by the European Breast Guidelines screening recommendations; [10] thus, policy makers should note that we did not include modelling estimates for women between the ages of 40 and 44 as screening is not suggested in this age group [10]. Also, readers should be careful when interpreting the effects of screening intervals across the different age groups, as comparisons are limited by the small number of screening rounds in the 45 to 49 and 70 to 74 age groups, compared to the 50–69 age group.

Recommendations about mammography screening intervals will also depend on the magnitude and relative importance of potential harms. Narayan, et al. assessed to what extent harms should decrease in order to make a screening interval with an unfavourable balance of benefits and harms acceptable [61]. They found that for annual screening a reduction of 31% false positive results would be required to support a recommendation in favour of starting at 50, although this was in the context of false positive rates prevailing in the US [61]. Policy makers should probably consider implementing interventions to improve mammography performance, mitigating concerns about potential harms. For example previous studies suggest that comparing mammograms with prior exams can significantly reduce the recall rate while maintaining the same detection rate [62, 63].

Several research priorities were identified during this review, with feedback from the GDG experts, such as need for: (i) empirical research on the effectiveness of the different screening intervals due to the current very low certainty of evidence; ii) cost-effectiveness studies using unitary costs from different settings, and in particular for women aged 45 to 49, iii) assessment of alternative imaging modalities, iv) tailored screening according to risk vs population screening. For example, previous research has highlighted that breast density influences both mammography accuracy and risk of developing breast cancer [64, 65]. For further information on the complete recommendations formulated in the European Guidelines on Breast Cancer Screening and Diagnosis, please visit the ECIBC website (https://healthcare-quality.jrc.ec.europa.eu/european-breast-cancer-guidelines/screening-ages-and-frequencies).

## Disclaimer

All views expressed in this article are strictly those of the authors.

## Supplementary information

Reproducibility checklist

Supplementary material file

## Data Availability

All data sources used during this study are described in this published article and its additional information files. The datasets analysed are available from the corresponding author on reasonable request.
