# Supplementary material for: Benefits and harms of annual, biennial, or triennial breast cancer mammography screening for women at average risk of breast cancer: a systematic review for the European Commission Initiative on Breast Cancer (ECIBC)
Source: Br J Cancer. 2021 Nov 26;126(4):673–88. doi: 10.1038/s41416-021-01521-8 (PMC8854566; doi:10.1038/s41416-021-01521-8)
Supplement: Supplementary file 2 — Supplementary material file [file 41416_2021_1521_MOESM2_ESM.doc]

**Table S1. Protocol of the systematic review**

| **Question** | **Should an annual, biennial or triennial screening frequency be used for screening asymptomatic women?** |
| --- | --- |
| **Objective** | 1. To compare the benefits and harms of annual, biennial and triennial mammography screening interval for breast cancer in average-risk women in three age groups: 44 to 49, 50 to 69 and 70 to 74 years old. |
| **Eligibility criteria** | **Study designs:** we will initially include existing relevant systematic reviews of randomized controlled trials. To update the existing reviews or to develop a de novo evidence synthesis, in the absence of relevant systematic reviews, we will search for individual studies  If no randomized trials with direct comparison of different mammography screening intervals are available we will look for observational evidence, initially prioritizing existing relevant systematic reviews as above. We will include prospective or retrospective cohorts, with intervals of screening determined on at least two examinations prior to diagnosis, and also includes studies assessing the effect of changing a recommended interval for a given screening program (before-after).  We will also include modelling studies assessing two or more screening intervals with a no screening based case scenario |
|  | **Population:** women 45 to 74 years of average risk for breast cancer |
|  | **Intervention:** annual or biennial or triennial mammography screening (film or digital). |
|  | **Comparison:** other interval (annual or biennial or triennial) as comparison of mammography screening for observational studies, or no screening for modelling studies if at least two different intervals are included in the analysis. |
|  | Outcomes:  1. Breast cancer mortality 2. Incidence of interval cancer 3. Stage of breast cancer – assumes detecting cancer in stage I is better than stage II, better than stage III and better than stage IV, because the treatment is less invasive , there are less side effects and there is a better chance of survival 4. Adverse effects (including radiation exposure, radiation induced cancers-related to radiation dose, overdiagnosis related adverse effects, false positive related adverse effects) 5. Incidence of advanced cancer following first round of screening 6. Quality of life –includes Anxiety caused or relieved by screening, anxiety caused by assessment of suspicious screening findings, longer length of life qualified by longer periods of life spent with a diagnosis of breast cancer, treatment side effects including psychosocial effects of body image following surgery 7. Other cause mortality |
|  | **Limits**:  We will exclude:   1. Studies about women at high risk for breast cancer, defined as having known susceptibility gene mutation (BRCA1/BRCA2), history of previous breast cancer, history of lobular neoplasia, proliferative lesions on prior biopsy or chest irradiation (other than mammography screening). 2. Studies that address indirect comparisons 3. Studies published in English and available at full text (not conference reports) |
| **Search strategy** | **Sources:**  We will retrieve relevant literature implementing combination algorithms of controlled vocabulary and search terms in the following databases: i) MEDLINE (accessed through Ovid); ii) The Cochrane Library; iii) EMBASE (accessed through Ovid). We will adapt the search algorithms to the requirements of each database, and we will use validated filters to retrieve appropriate designs as needed.  In the case of systematic reviews we will not limit the search by the date of publication, searching each database from its inception. In case of individual studies, if relevant systematic reviews are identified, we will search since the date of the last search of those selected reviews. Otherwise we will include studies published since 2000 (considering secular trends in survival and diagnostic performance due to technology and treatment progress).  We will also review references of included studies and ask guideline panellists for additional relevant studies that could potentially fulfil our eligibility criteria. We will report, in appendices, the complete search algorithms designed for each database, the hits retrieved, and the reasons for the exclusion of studies at the full text stage. |
|  | **Data management:** we will use EndNote X6 software to create a database for the management of the search results. |
| **Study selection, evidence appraisal and synthesis** | **Study selection**: One reviewer will screen the search results based on the title and abstract. Another reviewer will cross check a random selection of 20% of the total number of hits. Two reviewers will independently confirm eligibility based on the full text of the relevant articles. In case of discordance, they will reach consensus by discussion or involving a third reviewer. We will report the result of this process with a PRISMA flowchart. |
|  | **Data collection:** one reviewer will extract relevant data from eligible studies on their main characteristics. We will describe in a table the reasons that led to the decision to exclude a study, and describe in tables the main characteristics of the included studies, outcomes of interest and their main effect estimates. A different researcher will cross check the data extracted for accuracy. |
|  | **Risk of bias:** we will assess bias using: i) Cochrane Risk of Bias tool for trials (Higgins 2011); ii) ROBINS-I tool for observational studies (Sterne JA 2016); and ii) The ISPOR-AMCP-NPC Questionnaire to assess relevance and credibility of modelling/simulation studies (Caro 2014). |
|  | **Synthesis of the results:**  We will describe the effect estimates or main results obtained in narrative summaries (evidence profiles) according to the type of study design included in the review. When feasible we will conduct a pooled analysis applying the Mantel-Haenszel method under a random-effects model (Review Manager v 5.3) for relative risks, and for incidence or proportions we will use exact binomial statistic under *metaprop* procedure in Stata v.12. We will assess heterogeneity through the I2 statistic, and explore sources of variation between studies. |
|  | **Quality of the evidence:** we will rate the quality of evidence for each outcome of interest with GRADE. We will rate the quality of evidence across each outcome of interest as high, moderate, low or very low, depending on several factors including; risk of bias, imprecision, inconsistency, indirectness and publication bias. We will develop for each relevant comparison a GRADE evidence profile to reflect the judgements that allowed us to rate the quality of evidence for each outcome. |

**Table S2. Search strategy for the evidence of effects**

| **Clinical question**  **Should an annual, biennial or triennial screening frequency be used for screening asymptomatic women?** | |
| --- | --- |
| **MEDLINE**  PubMed  April 20, 2020 | #1 "Breast Neoplasms"[Mesh]  #2 breast[tiab]  #3 #1 OR #2  #4 "Mass Screening"[Mesh]  #5 "Early Detection of Cancer"[Mesh]  #6 screen*[ti]  #7 #4 OR #5 OR #6  #8 #3 AND #7  #9 "Mammography"[Mesh]  #10 mammogra*[ti]  #11 #8 OR #9 OR #10  #12 interval*[ti]  #13 screening interval*[tiab]  #14 year interval*[tiab]  #15 frequency[tiab]  #16 frequen*[ti]  #17 regular screening[tiab]  #18 timing[ti]  #19 annual*[ti]  #20 biennial*[ti]  #21 triennial*[ti]  #22 #12 OR #13 OR #14 OR #15 OR #16 OR #17 OR #18 OR #19 OR #20 OR #21 759474  #23 #11 AND #22  #24 “Models, Statistical”[Majr]  #25 model*[ti]  #26 simulation[ti]  #27 modeling[tiab]  #28 modelling[tiab]  #29 #24 OR #25 OR #26 OR #27 OR #28  #30 #11 AND #29  #31 #23 NOT #30  #32 systematic[sb]  #33 #31 AND #32  #34 #23 NOT (#30 OR #33)  #35 (randomized controlled trial[pt] OR controlled clinical trial[pt] OR randomized[tiab] OR placebo[tiab] OR drug therapy[sh] OR randomly[tiab] OR trial[tiab] OR groups[tiab]) NOT (animals [mh] NOT humans [mh]) 3353129  #36 #34 AND #35 384  #37 #23 NOT (#30 OR #33 OR #36)  #38 “Cohort Studies”[Mesh]  #39 “Time Factors”[Mesh]  #40 “Prospective Studies”[Mesh]  #41 “Regression Analysis”[Mesh:NoExp]  #42 “Logistic Models”[Mesh]  #43 cohort*[tiab]  #44 observational[tiab]  #45 prospective*[tiab]  #46 register*[tiab]  #47 registry[tiab]  #48 registries[tiab]  #49 regression[tiab]  #50 #38 OR #39 OR #40 OR #41 OR #42 OR #43 OR #44 OR #45 OR #46 OR #47 OR #48 OR #49  #51 #37 AND #50  #52 #30 OR #33 OR #36 OR #51 |
| **The Cochrane Library**  April 20, 2020 | #1 MeSH descriptor: [Breast Neoplasms] explode all trees  #2 breast:ti,ab  #3 #1 or #2 2532  #4 MeSH descriptor: [Mass Screening] explode all trees  #5 MeSH descriptor: [Early Detection of Cancer] explode all trees  #6 screen*:ti  #7 #4 or #5 or #6  #8 #3 and #7  #9 MeSH descriptor: [Mammography] explode all trees 1019  #10 mammogra*:ti  #11 #8 or #9 or #10  #12 interval*:ti  #13 (screening near/3 interval*):ti,ab  #14 (year near/3 interval*):ti,ab  #15 frequency:ti,ab  #16 frequen*:ti  #17 (regular near/3 screening):ti,ab  #18 timing:ti  #19 annual*:ti  #20 biennial*:ti  #21 triennial*:ti  #22 #12 or #13 or #14 or #15 or #16 or #17 or #18 or #19 or #20 or #21  #23 #11 and #22 |
| **EMBASE**  Ovid Embase April 20, 2020 | 1 *breast tumour/  2 breast.ti.  3 1 or 2  4 *screening/  5 *early diagnosis/  6 screen*.ti.  7 4 or 5 or 6  8 3 and 7  9 exp mammography/  10 mammogra*.ti.  11 8 or 9 or 10  12 interval*.ti.  13 (screening adj3 interval*).ti,ab.  14 year interval*.ti,ab.  15 frequency.ti,ab.  16 frequen*.ti.  17 (regular* adj3 screening).ti,ab.  18 timing.ti.  19 annual*.ti.  20 biennial*.ti.  21 triennial*.ti.  22 12 or 13 or 14 or 15 or 16 or 17 or 18 or 19 or 20 or 21  23 *statistical model/  24 model*.ti.  25 simulation.ti.  26 modeling.ti.  27 modelling.ti.  28 23 or 24 or 25 or 26 or 27  29 11 and 28  30 11 and 22  31 30 not 29  32 limit 31 to "systematic review"  33 31 not 32  34 random:.tw. or placebo:.mp. or double-blind:.mp.  35 33 and 34  36 33 not 35  37 Cohort Studies/  38 exp time factor/  39 Prospective Studies/  40 exp regression analysis/  41 cohort*.ti,ab.  42 observational.ti,ab.  43 prospective*.ti,ab.  44 register*.ti,ab.  45 registry.ti,ab.  46 registries.ti,ab.  47 regression.ti,ab.  48 37 or 38 or 39 or 40 or 41 or 42 or 43 or 44 or 45 or 46 or 47  49 36 and 48  50 29 or 32 or 35 or 49 |

**Table S3a. Overview of included primary studies on average risk women.**

| **Study ID** | **Design** | **Sample** | **All-cause mortality and Breast cancer mortality**  **(95%CI)** | **Advances stage**  **(95%CI)** | **Interval cancer**  **(95%CI)** | **False positive**  **(95%CI)** | **Benign Biopsies**  **(95%CI)** |
| --- | --- | --- | --- | --- | --- | --- | --- |
| **Braithwaite, 2012** | Case series and cohort  Registries: BCSC, 4 mammography sites  Age: 66 to 89 years  Period: 1999-2006  Country: United States | Annual:1,227  Biennial: 453  FP-Cohort: 137,949 |  | 66-74 years  Advanced stage (≥IIB): RR 0.75 (0.46;1.22)  Tumour size>2cm: RR 0.83 (0.55-1.24)  Lymph nodes: RR 0.84 (0.57-1.23)  75-89 years  Advanced stage (≥IIB): RR 1.27 (0.72-2.25)  Tumour size>2cm: RR 1.30 (0.83-2.05)  Lymph nodes: RR 0.83 (0.51-1.33) | Proportion of breast cancer cases  66 to 89  Annual: 23% (22% to 25%)  Biennial: 33% (30% to 36%) | Probability after 10-years of screening  66-74 years  Annual: 49.7% (47.8%; 51.5)  Biennial: 30.2 (29.4; 31.1)  75-89 years  Annual: 47.2% (44.9%-49.5%)  Biennial: 26.6% (25.7%-27.5%) | Probability after 10-years of screening  66-74 years  Annual: 9.8% (8.4%; 11.3%)  Biennial: 4.6% (4.2%; 5.1%)  75-89 years  Annual: 9.2% (7.5%; 11.2%)  Biennial: 4.1% (3.7%; 4.6%) |
| **Coldman, 2008** | Post-intervention  50-79 years  1998-2005 vs pre-1997  Data from Screening Mammography Programme of British Columbia  Canada |  |  | Tumour size>2cm  1.08 (0.96-1.22)  Grade (poor): 0.97 (0.86-1.10)  Lymph nodes: 1.23 (1.07-1.40) | RR: 1.06 (0.97-1.16) |  |  |
| **Dittus, 2013** | Case series and cohort  Registries: BCSC, multisite  Age: 40- 74 years  Period: 1996-2008  Country: United States | Annual: 2,766  Biennial: 1,666  FP-Cohort: 555,343 |  | Postmenopausal women  No difference in stage, tumour size, or node positive across all BMI categories  Premenopausal women  No difference in stage, tumour size, or node positive across all BMI categories |  | Probability after 10-years of screening  Age 50-74  Annual: 52.1% to 54.4% across BMI  Biennial: 32.6% to 34.3% across BMI  Age 40-49  A: 60.8% to 66.5% across BMI  B: 39.9% to 44.8% across BMI | Probability after 10 years of screening  Age 50-74  Annual: 7.9& to 9.2% across BMI  Biennial: 4.6% to 5.4% across BMI  Age 40-49  A: 11.2% to 11.5% across BMI  B: 6.0% to 6.2% across BMI |
| **Duffy, 2008 (Breast Screening Frequency Trial)*** | Randomized clinical trial  Age: 50-62 years  Period: 1989-2006  Country: United Kingdom | Per protocol  Annual: 37,530  Triennial: 38,492 | Age 50-62  RR = 0.89 (0.73-1.07, p = 0.2). |  |  |  |  |
| **Goel,**  **2007** | Case series  Vermont Breast  Cancer Surveillance  System  Period: 1994–2002  >40 years  Country: United States | Annual: 1,236  Biennial: 439 |  | 40–49 years  women (28.3% vs. 28.8%,  50-64 years  p=0.925), women 50–64 (19.2% vs. 22.3%, p=0.404) and  women 65 and older (19.8% vs.  20.8%, p=0.780). |  |  |  |
| **Hubbard, 2011** | Case series and cohort  Registries: BCSC, multisite  Age: 40- 59 years.  Period: 1994-2006  Country: United States | Cohort: 169,456 | --- | Difference in proportions  Age 40 to 49 years  Stage III/IV: 4.8 (1.3; 8.4)  Late stage (≥IIB): 3.3 (-1.1; 7.8)  50-59 years  Stage III/IV: 0.6 (-2.0; 3.1)  Late stage (≥IIB): 2.3 (-1.0; 5.7) | --- | Probability after 10-years of screening  Starting Age  Annual: 61.3% (59.4%; 63.1%)  Biennial: 41.6% (40.6%; 42.5%)  Age 50  Annual: 61.3% (58.0%; 64.7%)  Biennial: 42.0% (40.4%; 43.7%) | Probability after 10-years of screening  Age 40  Annual: 7.0% (6.1%; 7.8%)  Biennial: 4.8% (4.4%; 5.2%)  Age 50  Annual, 9.4% (7.4%; 11.5%)  Biennial, 6.4% (5.6%; 7.2%) |
| **Hunt,**  **1999** | Retrospective cohort  Age 40-79 years  Period: 1985- 1997  Country: United States | Annual: 19,905  Biennial: 4,306 | --- | Stage II (lymph positive)  Annual: 15%  Biennial: 21% | Annual: 0.07%  Biennial: 0.15% | --- | --- |
| **Kerlikowske, 2013** | Case series and cohort  Registries: BCSC, 7 mammography sites  Age: 40-74 years  Period: 1994-2008  Country: United States | Annual: 7,039  Biennial: 3,476  Triennial: 959  FP-Cohort: 922,624 | --- | 50-74 years  Advanced stage (≥IIB)  2-y/1-y: OR 0.74 (0.24; 2.28)  3-y/2-y: OR 1.05 (0.20; 5.50)  Tumour size>2cm  2-y/1-y: OR 1.36 (0.51-3.62)  3-y/2-y: OR 0.55 (0.12-2.47)  Lymph nodes  2-y/1-y: OR 0.75 (0.30-1.88)  3-y/2-y: OR 0.43 (0.08-2.31)  40-49 years  Advanced stage (≥IIB)  2-y/1-y: OR 0.76 (0.44-1.33)  3-y/2-y: OR 0.99 (0.45-2.18)  Tumour size>3.2cm  2-y/1-y: OR 0.88 (0.55-1.41)  3-y/2-y: OR 1.27 (0.67-2.40)  Lymph nodes:  2-y/1-y: OR 1.19 (0.75-1.89)  3-y/2-y: OR 0.95 (0.50-1.78) | Proportion of breast cancer cases  40 to 74  Annual: 30% (29% to 31%)  Biennial: 41% (39% to 42%)  Triennial: 44% (41% to 48%) | Probability after 10-years of screening  40-49 years  Annual: 60.0% (58.6%; 61.3%)  Biennial: 38.5% (37.8%; 39.3%)  Triennial: 27.0% (26.3; 27.6%)  50-74 years  Annual: 49.8% (49.0%; 50.6%)  Biennial: 30.7% (30.2%; 31.2%)  Triennial: 21.9% (21.3%; 22.4%) | Probability after 10-years of screening  40-49 years  Annual: 9.3% (8.3%; 10.4%)  Biennial: 4.9% (4.6%; 5.3%)  Triennial: 3.4% (3.1%; 3.7%)  50-74 years  Annual: 8.1% (7.6%; 8.6%)  Biennial: 4.5% (4.3%; 4.8%)  Triennial: 3.4% (3.2%; 3.7%) |
| **Klemi, 1997** | Quasi-experimental  Age: 40-49 years  Period: 1987-2003  Country: Finland | Triennial: 6,926  Annual: 7,839 (ref) | --- | --- | Proportion of breast cancer cases  40 to 49  Annual screening 25% (15% to 36%)  Triennial screening 35% (22% to 50%) | --- | --- |
| **Miglioretti, 2015** | Case series  Registries: BCSC  Age: 40-85 years  Period: 1996-2012  Country: United States | Annual: 12,070  Biennial: 3,370 |  | 40-49 years  Advanced stage (>IIB): RR 1.17 (0.93-1.46)  Tumour (>1.5cm): RR 1.10 (0.98-1,29)  Lymph node: RR 1.09 (0.92-1.29)  50-59 years  Advanced stage (>IIB): RR 0.98 (0.80-1.21)  Tumour (>1.5cm): RR 1.09 (0.97-1.21)  Lymph node: RR 1.05 (0.90-1.22)  60-69 years  Advanced stage (>IIB): RR 0.99 (0.79-1.24)  Tumour (>1.5cm): RR 1.13 (1.00-1.27)  Lymph node: RR 0.93 (0.78-1.12)  70-85 years  Advanced stage (>IIB): RR 0.98 (0.76-1.27)  Tumour (>1.5cm): RR 1.13 (0.99-1.29)  Lymph node: RR 0.91 (0.74-1.12) | Proportion of breast cancer cases  40 to 85  Annual: 22.2%  Biennial: 27.2% | Case series  Registries: BCSC  Age: 40-85 years  Period: 1996-2012  Country: United States | Annual: 12,070  Biennial: 3,370 |
| **McGuinnes.**  **2019** | Retrospective cohort  Age: all age (80% >50 years)  Period: 2014 -2015 (recruitment)  Country: United States |  |  |  |  | All ages (80% >50 years)  Annual: 59.7% (57.1% to 62.3%)    Biennial: 41.5% (36.2% to 47%) |  |
| **O’Meara, 2013** | Case series and cohort  Registries: BCSC, multisites  Age: 50-74 years  Period: 1994-2008  Country: United States | Annual: 8,876  Biennial: 4,265  Triennial: 1,255  FP-cohort: 1,276,312 |  | 40-49 years  Advanced stage(≥IIB):  2-y/1-y: OR 1.14 (0.89; 1.46)  3-y/2-y: OR 0.78 (0.54; 1.11)  Tumour size>2cm  2-y/1-y: OR 1.14 (0.91; 1.43)  3-y/2-y: OR 0.96 (0.70; 1.32)  Lymph nodes  2-y/1-y: OR 1.19 (0.95-1.48)  3-y/2-y: OR 0.97 (0.71; 1.32)  50-74 years  Advanced stage  2-y/1-y: OR 1.03 (0.89-1.19)  3-y/2-y: OR 0.83 (0.65; 1.07)  Tumour size>2cm  2-y/1-y: OR 1.12 (0.99-1.27)  3-y/2-y: OR 1.15 (0.93; 1.41)  Lymph nodes:  2-y/1-y: OR 1.04 (0.92-1.18)  3-y/2-y: OR 0.98 (0.80; 1.21) | --- | 40-49 years  Annual: 64.5% (63.3%; 65.4%)  Biennial: 41.1% (40.7%; 41.6%)  Triennial: 29.2% (28.8%; 29.6%)  50-74 years  Annual: 55.2% (54.8%; 55.7%)  Biennial: 35.4% (35.0%; 35.7%)  Triennial: 24.8% (24.5%; 25.2%) | 40-49 years  Annual: 11.4% (10.5%; 12.4%)  Biennial: 5.9% (5.6%; 6.2%)  Triennial: 3.9% (3.7%; 4.1%)  50-74 years  Annual: 9.7% (9.3%; 10.1%)  Biennial: 5.4% (5.2%; 5.6%)  Triennial: 3.7% (3.6%; 3.9%) |
| **Parvinen, 2011** | Quasi-experimental  Age: 40-49 years  Period: 1987-2003  Country: Finland | Triennial: 6,926  Annual: 7,839 (ref) | Age 40-49  All-cause  RR: 1.20 (0.99 – 1.46)  Breast cancer  RR: 1.14 (0.59 – 1.27) |  |  |  |  |
| **Sanderson, 2015** | Cohort  Registries: data from Medicare claims and SEER  Age: 69-84 years  Period: 1995-2000  Country: United States | n=64,384 | Breast Cancer  10 year mortality after diagnosis (No or irregular screening as reference)  Biennial: HR 0.47 (0.44-0.51)  Annual: HR 0.31 (0.29-0.33)  *p value* for trend: <0.001 | --- | --- | --- | --- |
| **White, 2004** | United States: BCSC data,  Multisite  Age: 40–89  Period: 1996–2001 |  |  | 40-49 years  Late stage: OR 1.37 (1.00 to 1.86)  Tumour size >2cm: OR 1.21 (0.88 to 1.67)  Grade III or IV: OR 1.05 (0.77 to 1.44)  50-59 years  Late stage: OR 0.93 (0.71 to 1.22)  Tumour size >2cm>: 1.00 (0.76 to 1.32)  Grade III or IV: 0.81 (0.61 to 1.08)  60-69 years  Late stage 0.90 (0.65 to 1.23)  Tumour size _ 20 mm 0.88 (0.63 to 1.22)  Grade III or IV 1.16 (0.85 to 1.57)  70-79 years  Late stage: OR 0.80 (0.58 to 1.10)  Tumour size >2cm: OR 1.20 (0.89 to 1.61)  Grade III or IV: OR 1.07 (0.81 to 1.42) | Proportion of breast cancer cases  40 to 89  Annual: 26%  Biennial: 38% |  |  |
| *Congress abstract. Duffy et al. presented update results of the UK Breast Screening Frequency Trial on actual breast cancer mortality until the end of 2006 | | | | | | | |

**Table S3b. Overview of included modelling studies on average risk women**

| **Study ID** | **Model** | **Breast cancer deaths averted** | **False positive** | **Benign Biopsies** | **Over-diagnosis** | **Radiation induced breast cancer** | **Death by radiation induced breast cancer** | **QUALYs** |
| --- | --- | --- | --- | --- | --- | --- | --- | --- |
| **Arnold,**  **2019** | Microsimulaiton Markov model  n= 3,000,000  Time Horizon: death or 100 years  Country: Germany | --- | --- | 50-69 years  Annual: 9,600  Biennial: 5,000  Triennial: 3,700 | --- | --- | --- | 50-69 years  Annual: 4,400  Biennial: 3,900  Triennial: 3,300 |
| **Gunsoy,**  **2014** | Markov model  n=10,000  Time Horizon: 85 years old, starting at age 40  Country: United Kingdom | 40-73 also analyzed  47-73 years  Annual: 900 (880 to 920)  Triennial: 470 (440 to 490) | --- | --- | 47-73 years  Annual: 800 (750 to 850)  Triennial: 580 (530 to 630) | --- | --- | --- |
| **Madelblatt, 2016** | Model D  Model E  Model GE  Model M  Model S  Model W  Time Horizon: Lifetime  n=1,000  Contry: United States | 40-49 years  Annual: 130  Biennial: 100  45-49 years*  Annual: 70  Biennial: 40  50-74 years  Annual: 900  Biennial: 700 | 40-49 years*  Annual: 115,300  Biennial: 57,600  45-49 years  Annual: 56,700  Biennial: 26,700  50-74 years  Annual: 179,800  Biennial: 95,300 | 40-49 years*  Annual: 11,000  Biennial: 6,700  45-49 years  Annual: 5,600  Biennial: 3,000  50-74 years  Annual: 22,800  Biennial: 14,600 | 40-49 years*  Annual: 400  Biennial:200  45-49 years  Annual: 200  Biennial: 0  50-74 years  Annual: 2,500  Biennial:1,900 | --- | --- | 45-49 years  Annual: 1,540  Biennial: 1,060  40-49 years*  Annual: 2,330  Biennial:1,860  50-74 years  Annual: 9,950  Biennial:8,600 |
| **Miglioretti, 2016** | Radiation Exposure Model  n=100,000  Time Horizon: Lifetime  Country: United States | 45-49 years*  Annual: 90  Biennial: 39  40-49 years*  Annual: 153  Biennial: 105  50-74 years  Annual: 819  Biennial: 627 | --- | --- | --- | 45-49 years  Annual: 32  Biennial: 18  40-49 years*  Annual: 62  Biennial: 41  50-74 years  Annual: 49  Biennial: 27 | 45-49 years  Annual: 6  Biennial: 4  40-49 years*  Annual: 11  Biennial: 8  50-74 years  Annual: 7  Biennial: 4 | --- |
| **Mittman,** | Model W  n= 2,000,000  Time Horizon: Lifetime  Country: Canada | --- | --- | --- | --- | --- | --- | 50-69 years  Annual: 7,100  Biennial: 5,000  Triennial: 3,800  70-74 years  Annual: 600  Biennial: 500  Triennial: 300 |
| **Tsunematsu, 2015** | Math transition model  n=100,000  Time Horizon: Lifetime  Country: Japan/ United States | 40-49 years*  Annual: 175  Biennial: 131  50-69 years  Annual: 870  Biennial: 705 | 40-49 years*  Annual: 95,226  Biennial: 47,692  50-69 years  Annual: 114,430  Biennial: 57,470 | --- | --- | --- | --- | --- |
| **Trentham-Dietz, 2016**** | Model E  Model G-E  Model W  n=1,000  Time Horizon: 25 to 100 years  Country: United States | 50-74 years  Annual: 870  Biennial: 650  Triennial: 510 | 50-74 years  Annual: 177,600  Biennial: 99,400  Triennial: 76,700 | 50-74 years  Annual: 20,600  Biennial: 14,800  Triennial: 12,300 | 50-74 years  Annual: 2,900  Biennial: 2,000  Triennial: 1,600 | --- | --- | 50-74 years  Annual: 8,200  Biennial: 6,300  Triennial:  4,800 |
| **Van Ravesteyn, 2012** | Model D  Model E  Model G-E  Model W  Time Horizon: Lifetime  n=1,000  Country: Unites States | 40-49 years  Annual: 170  Biennial: 120 | 40-49 years  Annual: 117,500  Biennial: 59,200 |  |  |  |  |  |
| **Vilaprinyo, 2014** | Transition model  n=100,000  Country: Spain | 40-49 years  Annual: 53  Biennial: 37  Triennial: 5  45-49 years  Annual: 33  Biennial: 52 (end at 69 yo); 7 (ends at 74 yo)  Triennial:  47 (end at 69 yo); -16 (end at 74 yo)  50-69 years  Annual: 631  Biennial: 426  Triennial: 397  50-74 years  Annual: 773  Biennial: 571  Triennial: 533  70-74 years  Annual: 142  Biennial: 145  Triennial: 156 | 40-49 years  Annual: 19,448  Biennial: 12,509  Triennial: 9,532 (end at 69 yo); 9,554 (end at 74 yo)  45-49 years  Annual: 9,150  Biennial: 6,301 (end at 69 yo); 5,149 (end at 74 yo)  Triennial: 4,831 (end at 69 yo); 3,698 (end at 74 yo)  50-69 years  Annual: 42,606  Biennial: 29,039  Triennial: 24,252  50-74 years  Annual: 48,372  Biennial: 32,498  Triennial: 26,547  70-74 years  Annual: 5,766  Biennial: 3,459  Triennial: 2,295 | 40-49 years  Annual: 919  Biennial: 428 (end at 69 yo); 398 (end at 74 yo)  Triennial: 229  45-49 years  Annual: 409  Biennial: 208 (end at 69 yo); 98 (end at 74 yo)  Triennial: 108 (end at 69 yo); 23 (end at 74 yo)  50-69 years  Annual: 3,455  Biennial: 2,487  Triennial: 2,166  50-74 years  Annual: 3,883  Biennial: 2,774  Triennial: 2,337  70-74 years  Annual: 428  Biennial: 287  Triennial: 171 | 40-49 years  Annual: 266  Biennial: 147  Triennial: 72 (end at 69 yo); 63 (end at 74 yo)  45-49 years  Annual: 143  Biennial: 119 (end at 69 yo); 41 (end at 74 yo)  Triennial: 88 (end at 69 yo); -9 (end at 74 yo)  50-69 years  Annual: 904  Biennial: 609  Triennial: 500  50-74 years  Annual: 1,173  Biennial: 845  Triennial: 693  70-74 years  Annual: 269  Biennial: 236  Triennial: 193 |  |  | 40-49 years  Annual: 1197  Biennial: 863  Triennial: 652 (end at 69 yo); 780 (end at 74 yo)  45-49 years  Annual: 727  Biennial: 665 (end at 69 yo); 526 (end at 74 yo)  Triennial: 653 (end at 69 yo); 432 (end at 74 yo)  50-69 years  Annual: 6,801  Biennial: 4,714  Triennial: 4,386  50-74 years  Annual: 7,137  Biennial: 5,141  Triennial: 4,784  70-74 years  Annual: 336  Biennial: 427  Triennial: 398 |
| **Yaffe, 2011** | Model by Preston  n=100,000  Time Horizon: 85 years old  Country: Canada | --- | --- | --- | --- | 50-59 years  Annual: 27  Biennial: 14 | 50-59 years  Annual: 3.1  Biennial: 1.6 | --- |
| **Yaffe, 2015** | CISNET model  n=1,000  Time Horizon: lifetime  Country: Canada | 40-49 years  Annual: 180  Biennial: 120  50-69 years  Annual: 740  Biennial: 520  Triennial: 400  70-74 years  Annual: 100  Biennial: 90  Triennial: 80 | 40-49 years*  Annual: 101,100  Biennial: 62,900  50-69 years  Annual: 152,800  Biennial: 89,500  Triennial: 69,900  70-74 years  Annual: 24,500  Biennial: 17,400  Triennial: 12,700 | 40-49 years*  Annual: 11,400  Biennial: 9,700  50-69 years  Annual: 16,300  Biennial:14,400  Triennial:14,100  70-74 years  Annual: 3,200  Biennial: 3,500  Triennial: 3,200 | --- | --- | --- | --- |
| *Effects on the age group 40 to 49 year for some studies were not directly reported by authors and were estimated by calculating the difference between the two overlapping groups (i.e. effects in 40 to 74 years minus effects in 50 to 74 years).  ** We extracted data only from the group of women with scattered firbroglandular breast density | | | | | | | | |

## Table S4: Reasons for exclusion

| **EVIDENCE OF EFFECT STUDIES** | |
| --- | --- |
| **Systematic reviews** | |
| **Study ID** | **Reason for exclusion** |
| 1. Harstall 2000 | Not all outcomes included |
| 1. Nelson 2016a | Not all outcomes included |
| 1. Nelson 2016b | Not all outcomes included |
| 1. Myers 2015 | Not all outcomes included |
| 1. Walter 2014 | Not all outcomes included |
| **Individual studies** | |
| **Study ID** | **Reason for exclusion** |
| 1. Blnachard 2006 | No acceptable definition for intervals |
| 1. Carlson 1999 | Not included because different age group or no direct comparison |
| 1. Chang 2016 | Report of adherence to screening. |
| 1. Chen 2010 | Modelling not including outcomes of interest |
| 1. Duffy 2003 | Update analysis without difference of first publication |
| 1. Elmore 1998 | Not included because different age group or no direct comparison |
| 1. Falk 2016 | No comparison of screening intervals |
| 1. Field 1998 | Not included because different age group or no direct comparison |
| 1. Friedman 2013 | No acceptable definition for intervals |
| 1. Gunsoy 2012 | No comparison of screening intervals |
| 1. Habtes 2013 | No comparison of screening intervals |
| 1. Hanin 2013 | No comparison of screening intervals |
| 1. Houssami 2013 | No comparison of screening intervals |
| 1. Jiang 2016 | No comparison of screening intervals |
| 1. Mandelblatt 2009 | An update report was published in 2016 |
| 1. Mandelblatt 2003 | No comparison of screening intervals |
| 1. Mandelblatt 2011 | An update report was published in 2016 |
| 1. Moss 2015 | No comparison of screening intervals |
| 1. Nystrom 2016 | No comparison of screening intervals |
| 1. O’Mahony 2015 | Validation model study. No comparison of screening intervals |
| 1. Randall 2009 | Not average risk women |
| 1. Roder 20008 | No comparison of screening intervals |
| 1. Roman 2013 | No comparison of screening intervals |
| 1. Seigneurim 2016 | No comparison of screening intervals |
| 1. Seigneurim 2015 | No comparison of screening intervals |
| 1. Simon 2014 | No acceptable definition for intervals |
| 1. Soon 2009 | No comparison of screening intervals |
| 1. Van Ravesteyn 2010 | An update report was published in 2011 |
| 1. Van Ravesteyn, 2012 | Simulation of results from a specific trial (actual data already available) |
| 1. Wai 2005 | Predicted mortality, no actual observations. |
| 1. Yaffe 2015 | Validation model study. No comparison of screening intervals |
| 1. Yankaskas 2005 | No acceptable definition for intervals |

**Evidence profiles**

**Table S5. Question**: Annual compared to biennial mammography for women 45-49 years

| **Certainty assessment** | | | | | | | **№ of patients** | | **Effect** | | **Certainty** | **Importance** |
| --- | --- | --- | --- | --- | --- | --- | --- | --- | --- | --- | --- | --- |
| **№ of studies** | **Study design** | **Risk of bias** | **Inconsistency** | **Indirectness** | **Imprecision** | **Other considerations** | **annual mammography screening** | **biennial mammography screening** | **Relative (95% CI)** | **Absolute (95% CI)** |  |  |
| **Breast cancer death averted** | | | | | | | | | | | | |
| 2 ^1,2,e^ | modelling studies | serious ^f,g^ | not serious | very serious ^h,i,j^ | not serious | none | 70 to 90 | 39 to 40 | **Ratio** 1.75 to 2.31 | **from 30 more to 51 more per 100.000** | ⨁◯◯◯ VERY LOW | CRITICAL |
| **Stage of breast cancer (IIB-IV)** | | | | | | | | | | | | |
| 1 ^3^ | observational studies | serious ^k^ | not serious | very serious ^c,l^ | not serious | none | 2052 cases 3573 controls | | **OR 0.85** (0.75 to 0.96) | - | ⨁◯◯◯ VERY LOW | CRITICAL |
|  |  |  |  |  |  |  | - | 0.0% |  | **--** |  |  |
| **QALYs** | | | | | | | | | | | | |
| 2 ^1,5, m^ | modelling studies | not serious | not serious | very serious ^h,i,j^ | not serious | none | 727 to 1,540 | 665 to 1,060 | **Ratio** 1.09 to 1.45 | **62 more to 480 more per 100.000** | ⨁◯◯◯ VERY LOW | CRITICAL |
| **Interval cancer** | | | | | | | | | | | | |
| 1 ^4,n^ | observational studies | serious ^k^ | serious | very serious ^o^ | not serious | none | 10/14285 (0.1%) | 5/3333 (0.2%) | **RR 0.46** (0.16 to 1.36) | **81 fewer per 100.000** (from 126 fewer to 54 more) | ⨁◯◯◯ VERY LOW | CRITICAL |
| **Overdiagnosis** | | | | | | | | | | | | |
| 2 ^1,5,m^ | modelling studies | not serious | not serious | serious ^h,i,j^ | not serious | none | 143 to 200 | 0 to 119 | **Ratio :Not estimable to** 1.2 | **24 more to 200 more per 100.000** | ⨁◯◯◯ VERY LOW | CRITICAL |
| **False positive results -10 year cumulative probability^p^** | | | | | | | | | | | | |
| 1 ^6^ | observational studies | serious ^q^ | not serious | very serious ^c,l^ | not serious | none | Annual screening 67% (95%CI 65% to 68%) Biennial screening 45% (95%CI 44% to 46%) Difference: 22,000 more per 100,000. | | | | ⨁◯◯◯ VERY LOW | CRITICAL |
| **False positive biopsy recommendation -10 year cumulative probability^r^** | | | | | | | | | | | | |
| 1 ^6^ | observational studies | serious ^q^ | not serious | very serious ^c,l^ | not serious | none | Annual screening 11% (10% to 13%) Biennial screening 6% (5% to 7%) Difference: 5,000 more per 100,000. | | | | ⨁◯◯◯ VERY LOW | CRITICAL |
| **Radiation induce breast cancer** | | | | | | | | | | | | |
| 1 ^7,s^ | modelling studies | serious ^f,g^ | not serious | very serious ^h,i,j,t^ | not serious | none | 32 | 18 | **Ratio : 1.78** | **14 more per 100.000** | ⨁◯◯◯ VERY LOW | CRITICAL |
| **Death by radiation induced breast cancer** | | | | | | | | | | | | |
| 1 ^7,s^ | modelling studies | serious ^f,g^ | not serious | very serious ^h,i,j,t^ | not serious | none | 6 | 4 | **Ratio:1.5** | **2 more per 100.000** | ⨁◯◯◯ VERY LOW | CRITICAL |

**CI:** Confidence interval; **RR:** Risk ratio; **OR:** Odds ratio. For modelling studies, certainty of evidence starts from low certainty and when there is more than one study informing an outcome, the number represents the range of point estimates reported across studies.

#### Explanations

a. Rate ratio comparing annual screening relative to biennial screening was estimated by an indirect meta-analysis. Absolute effects were calculated taken as basal risk the proportion of breast cancer mortality in intervention arms of the trials of annual screening.

b. Comparison was done by performing indirect meta-analysis of RCT (n=3) of annual mammography interval versus no screening against RCT of biennial mammography interval versus no screening.

c. Estimations based on studies that included women from 40 to 49 years old

d. Wide confidence interval based in indirect comparison

e. Modelling studies used different number of women screened for calculations: 1,000 in 2 studies, and 100,000 in 2 studies. One modelling study (Vilaprinyo 2017) gave inconsistent results in this year period (less deaths averted for annual interval) and then it was not included in the results of breast cancer deaths averted.

f. One or more studies did not report information about external validation for the estimated parameters of the models.

g. One or more studies did not report sensitivity analysis information for the estimated parameters of the models.

h. The comparison for any interval in the models was a no screening scenario. No direct comparisons were reported.

i. Modelling studies with data available for the 45 to 49 age period. Results were calculated by subtracting the absolute number of events from overlapping periods of screening i.e. 45 to 74 minus 50 to 74.

j. Most models were constructed using data of surveillance registries from United States.

k. Intervals were classified based on the month ranges elapsed between two screening mammograms prior to diagnosis. Potential high risk of misclassification.

l. Results were extracted from groups of women with selected characteristics (e.g. normal weight, fatty or scattered fribroglandular breast density, or white race).

m. Modelling study, used 1,000 women screened for calculations.

n. From the In the Swedish two county trial with an average screening interval of 24 months, the calculated interval cancers for >0 to <12 months was 38%, and for 12 to <24 months was 68% (Tabar 1987).

o. Estimations based on one study that included women from 40 to 79 years old

p. Two modelling studies estimated the number of false positive results in annual screening of 9,150 to 56,700 and for biennial of 6,301 to 26,700 per 100,000 screened women from 45 to 49 years old (difference 2,849 to 30,000 more events).

q. No clear information of how the intervals were estimated for the false positive cohorts or the number of individuals per interval.

r. Two modelling studies estimated the number of benign biopsy results in annual screening of 409 to 5,600 and for biennial of 208 to 3,000 per 100,000 screened women from 45 to 49 years old (difference 201to 2,600 more events).

s. Modelling study, used 100,000 women screened for estimates.

t. Incremental effects were estimated for a screening program starting at 50 and ending at 74.

#### References

1. Mandelblatt JS, Stout NK Schechter CB van den Broek JJ Miglioretti DL Krapcho M Trentham-Dietz A Munoz D Lee SJ Berry DA van Ravesteyn NT Alagoz O Kerlikowske K Tosteson AN Near AM Hoeffken A Chang Y Heijnsdijk EA Chisholm G Huang X Huang H Ergun MA Gangnon R Sprague BL Plevritis S Feuer E de Koning HJ Cronin KA.. Collaborative modeling of the benefits and harms associated with different U.S. breast cancer screening strategies. Ann Intern Med; 2016.

2. Miglioretti, J. Radiation-induced breast cancer incidence and mortality from digital mammography screening.. Ann Intern Med; 2016.

3. Miglioretti DL, Zhu W,Kerlikowske K,Sprague BL,Onega T,Buist DS,Henderson LM,Smith RA, Consortium., Breast,Cancer,Surveillance. Breast tumor prognostic characteristics and biennial vs annual mammography, age, and menopausal status. JAMA Oncol; 2015.

4. Hunt, KA. Outcome analysis for women undergoing annual versus biennial screening mammography: a review of 24,211 examinations. AJR Am J Roentgenol; 1999.

5. Vilaprinyo, E. Cost-effectiveness and harm-benefit analyses of risk-based screening strategies for breast cancer. PLoS One; 2014

6. Dittus, K. Impact of mammography screening interval on breast cancer diagnosis by menopausal status and BMI. J Gen Intern Med ;

7. Miglioretti DL. Radiation-Induced Breast Cancer Incidence and Mortality From Digital Mammography Screening: A Modeling Study. Annals of internal medicine. 2016.

**Table S6. Question**: Triennial mammography screening compared to biennial mammography screening for early detection of breast cancer in women aged 45 to 49

| **Certainty assessment** | | | | | | | **№ of patients** | | **Effect** | | **Certainty** | **Importance** |
| --- | --- | --- | --- | --- | --- | --- | --- | --- | --- | --- | --- | --- |
| **№ of studies** | **Study design** | **Risk of bias** | **Inconsistency** | **Indirectness** | **Imprecision** | **Other considerations** | **triennial mammography screening** | **biennial mammography screening** | **Relative (95% CI)** | **Absolute (95% CI)** |  |  |
| **Breast cancer death averted** | | | | | | | | | | | | |
| 1 ^1,a,b^ | modelling studies | not serious | not serious | very serious ^c,d^ | not serious | none | 47 | 52 | **Ratio 0.9** | **5 fewer per 100.000** | ⨁◯◯◯ VERY LOW | CRITICAL |
| **Stage of breast cancer (IIB-IV)** | | | | | | | | | | | | |
| 1 ^2^ | observational studies | serious ^e^ | not serious | very serious ^f,g^ | not serious | none | 0 cases 0 controls | | **OR 0.78** (0.54 to 1.11) ^h^ | - | ⨁◯◯◯ VERY LOW | CRITICAL |
|  |  |  |  |  |  |  | - | 0.0% |  | **--** |  |  |
| **QALYs** | | | | | | | | | | | | |
| 1 ^1,a^ | modelling studies | not serious | not serious | very serious ^c,d^ | not serious | none | 653 | 665 | **Ratio 0.89** | **12 fewer per 100.000** | ⨁◯◯◯ VERY LOW | CRITICAL |
| **Overdiagnosis** | | | | | | | | | | | | |
| 1 ^1,a^ | modelling studies | not serious | not serious | very serious ^c,d^ | not serious | none | 88 | 119 | **Ratio 0.74** | **31 fewer per 100.000** | ⨁◯◯◯ VERY LOW | CRITICAL |
| **False positive -10 year cumulative probability** | | | | | | | | | | | | |
| 1 ^2^ | observational studies ^i^ | serious ^j^ | not serious | very serious ^k,l^ | not serious | none | Triennial screening 30% (95%CI 29% to 30%) Biennial screening 41% (95%CI 41% to 42%) Difference: 11,000 more per 100,000. | | | | ⨁◯◯◯ VERY LOW | CRITICAL |
| **False positive biopsy recommendation -10 year cumulative probability** | | | | | | | | | | | | |
| 1 ^2^ | observational studies ^m^ | serious ^n^ | not serious | very serious ^k,l^ | not serious | none | Triennial screening 4% (4% to 4%) Biennial screening 6% (6% to 6%) Difference: 2,000 more per 100,000. | | | | ⨁◯◯◯ VERY LOW | CRITICAL |
| **Interval cancer - not reported** | | | | | | | | | | | | |
| - | - | - | - | - | - | - | - | - | - | - | - | CRITICAL |
| **Incidence of advanced breast cancer following first round of screening - not reported** | | | | | | | | | | | | |
| - | - | - | - | - | - | - | - | - | - | - | - | CRITICAL |

**CI:** Confidence interval; **OR:** Odds ratio. For modeling studies, certainty of evidence starts from low certainty and when there is more than one study informing an outcome, the number represents the range of point estimates reported across studies.

#### Explanations

a. 100,000 women screened for calculations.

b. One quasi-experimental study from Finland did not report an incremental risk on breast cancer mortality from triennial vs annual interval in the 40 to 49 years age group (RR 0.88; 95%CI 0.43 to 1.79).

c. The comparison for any interval in the models was a no screening scenario. No direct comparisons were reported.

d. In the publications the age range 45-49 was not included. Results were calculated by subtracting the effects of overlapping age groups.

e. Intervals were classified based on the month ranges elapsed between two screening mammograms prior to diagnosis. Potential high risk of misclassification.

f. Estimations based on studies that included women from 40 to 49 years old.

g. Results were extracted from groups of women with selected characteristics (e.g. normal weight, fatty or scattered fibroglandular breast density, or white race).

h. Odds ratio for white women in the group of 40 to 49 years old and adjusted by age in years.

i. One modelling study estimated the number of false positive results in triennial screening of 4,831 and for biennial of 6,301 per 100,000 screened women from 45 to 59 years old (difference of 1,470 fewer events).

j. Intervals were classified based on the month ranges elapsed between two screening mammograms prior to diagnosis. Potential high risk of misclassification.

k. Estimations based in studies that included women from 40 to 49 years old

l. Results were extracted from groups of women with selected characteristics (e.g. normal weight, fatty or scattered fibroglandular breast density, or white race).

m. One modelling study estimated the number of benign biopsy results in triennial screening of 100 and for biennial of 6,301 per 100,000 screened women from 45 to 59 years old (difference of 100 fewer events).

n. Intervals were classified based on the month ranges elapsed between two screening mammograms prior to diagnosis. Potential high risk of misclassification.

#### References

1. Vilaprinyo, E. Cost-effectiveness and harm-benefit analyses of risk-based screening strategies for breast cancer. PLoS One; 2014.

2. O'Meara ES, Zhu W,Hubbard RA,Braithwaite D,Kerlikowske K,Dittus KL,Geller B,Wernli KJ,Miglioretti DL.. Mammographic screening interval in relation to tumour characteristics and false-positive risk by race/ethnicity and age. Cancer; 2013.

**Table S7 Question**: annual compared to triennial mammography screening for women aged 45 to 49

| **Certainty assessment** | | | | | | | **№ of patients** | | **Effect** | | **Certainty** | **Importance** |
| --- | --- | --- | --- | --- | --- | --- | --- | --- | --- | --- | --- | --- |
| **№ of studies** | **Study design** | **Risk of bias** | **Inconsistency** | **Indirectness** | **Imprecision** | **Other considerations** | **annual mammography screening** | **triennial mammography screening** | **Relative (95% CI)** | **Absolute (95% CI)** |
| **Breast cancer mortality** | | | | | | | | | | | | |
| 1 ^1^ | observational studies | not serious ^a,b^ | not serious | serious ^c^ | serious ^d^ | none | 18/88543 (0.0%) | 18/100508 (0.0%) | **RR 1.14** (0.59 to 2.19) | **3 more per 100.000** (from 7 fewer to 21 more) | ⨁◯◯◯ VERY LOW | CRITICAL |
| **Breast cancer death averted** | | | | | | | | | | | | |
| 1 ^2,e^ | modelling studies | not serious | not serious | very serious ^f,g^ | not serious | none | 33 | 47 | **Ratio 0.7** | **14 fewer per 100.000** | ⨁◯◯◯ VERY LOW | CRITICAL |
| **QALYs** | | | | | | | | | | | | |
| 1 ^2,e^ | modelling studies | not serious | not serious | very serious ^f,g^ | not serious | none | 727 | 653 | **Ratio 1.11** | **74 more per 100.000** | ⨁◯◯◯ VERY LOW | CRITICAL |
| **Overdiagnosis** | | | | | | | | | | | | |
| 1 ^2,e^ | modelling studies | not serious | not serious | very serious ^f,g^ | not serious | none | 142 | 88 | **Ratio 1.61** | **54 more per 100.000** | ⨁◯◯◯ VERY LOW | CRITICAL |
| **False positive results -10 year cumulative probability** | | | | | | | | | | | | |
| 1 ^3^ | observational studies ^h^ | serious ^i^ | not serious | very serious ^c,j^ | not serious | none | Annual screening 65% (95%CI 63% to 65%) Triennial screening 29% (95%CI 29% to 30%) Difference: 36,000 more per 100,000. | | | | ⨁◯◯◯ VERY LOW | CRITICAL |
| **False positive biopsy recommendation -10 year cumulative probability** | | | | | | | | | | | | |
| 1 ^3^ | observational studies ^k^ | serious ^i^ | not serious | very serious ^c,j^ | not serious | none | Annual screening 11% (11% to 12%) Triennial screening 4% (4% to 4%) Difference: 7,000 more per 100,000. | | | | ⨁◯◯◯ VERY LOW | CRITICAL |
| **Stage of breast cancer (IIB-IV) - not reported** | | | | | | | | | | | | |
| - | - | - | - | - | - | - | - | - | - | - | - | CRITICAL |
| **Interval cancer** | | | | | | | | | | | | |
| 1 ^4^ | observational studies | not serious ^a^ | not serious | serious ^d^ | not serious | none | Annual screening 25% (15% to 36%) Triennial screening 35% (22% to 50%) Difference: 10,000 fewer per 100,000 breast cancers | | | | ⨁◯◯◯ VERY LOW | CRITICAL |
| **Incidence of advanced breast cancer following first round of screening - not reported** | | | | | | | | | | | | |
| - | - | - | - | - | - | - | - | - | - | - | - | CRITICAL |

**CI:** Confidence interval; **RR:** Risk ratio. For modeling studies, certainty of evidence starts from low certainty and when there is more than one study informing an outcome, the number represents the range of point estimates reported across studies

#### Explanations

a. The study was carried out in the screening program of Turku-Finland, where since 1987, the women in the population age 40–49 years are screened annually (even year-of-birth cohorts) or triennially (odd birth-year cohorts). The analyses were performed according to the intention-to-screen.

b. Only deaths due to invasive breast cancers up to the age of 52 were included. We refer to this outcome as the incidence-based mortality, also referred to as refined mortality.

c. Estimations based on studies that included women from 40 to 49 years old.

d. The confidence interval of the point estimates goes from important benefit to important harms.

e. 100,000 women screened for calculations.

f. The comparison for any interval in the models was a no screening scenario. No direct comparisons were reported.

g. In the publications, the age range 45-49 was not included. Results were calculated by subtracting the effects of overlapping age groups.

h. One modelling study estimated the number of false positive results in annual screening of 9,150 and for triennial of 4,831 per 100,000 screened women from 45 to 59 years old (difference of 4,319 more events).

i. Intervals were classified based on the month ranges elapsed between two screening mammograms prior to diagnosis. Potential high risk of misclassification.

j. Results were extracted from groups of women with selected characteristics (e.g. normal weight, fatty or scattered fibroglandular breast density, or white race).

k. One modelling study estimated the number of benign biopsy results in annual screening to be 408 and for triennial screening to be 108 per 100,000 screened women from 45 to 59 years old (difference of 300 more events).

#### References

1. Parvinen I, Chiu S,Pylkkänen L,Klemi P,Immonen-Räihä P,Kauhava L,Malila N,Hakama M.. Effects of annual vs triennial mammography interval on breast cancer incidence and mortality in ages 40-49 in Finland. Br J Cancer; 2011.

2. Vilaprinyo, E. Cost-effectiveness and harm-benefit analyses of risk-based screening strategies for breast cancer. PLoS One; 2014.

3. O'Meara ES, Zhu W,Hubbard RA,Braithwaite D,Kerlikowske K,Dittus KL,Geller B,Wernli KJ,Miglioretti DL.. Mammographic screening interval in relation to tumour characteristics and false-positive risk by race/ethnicity and age. Cancer; 2013.

4. Klemi, P. Mammography screening interval and the frequency of interval cancers in a population-based screening. Br J Cancer; 1997.

**Table S8. Question:** Annual compared to biennial mammography for women 50-69 years

| **Certainty assessment** | | | | | | | **№ of patients** | | **Effect** | | **Certainty** | **Importance** |
| --- | --- | --- | --- | --- | --- | --- | --- | --- | --- | --- | --- | --- |
| **№ of studies** | **Study design** | **Risk of bias** | **Inconsistency** | **Indirectness** | **Imprecision** | **Other considerations** | **annual** | **biennial mammography screening** | **Relative (95% CI)** | **Absolute (95% CI)** |  |  |
| **Breast cancer mortality** | | | | | | | | | | | | |
| 1 ^1,a^ | observational studies | very serious ^b^ | not serious | not serious | not serious | none | 56/152226 (0.0%) | 128/184764 (0.1%) | **IRR 0.94** (0.68 to 1.31) | **4 fewer per 100.000** (from 22 fewer to 21 more) | ⨁◯◯◯ VERY LOW | CRITICAL |
| **Breast cancer death averted** | | | | | | | | | | | | |
| 3 ^2,3,4,a,c^ | modelling studies | not serious ^d,e^ | not serious | serious ^f,g^ | not serious | none | 631 to 870 | 426 to 705 | **Ratio**  1.23 to 1.48 | **165 more to 205 more** **per 100.000** | ⨁◯◯◯ VERY LOW | CRITICAL |
| **Stage of breast cancer (IIB-IV)** | | | | | | | | | | | | |
| 1 ^5^ | observational studies | serious ^h^ | not serious | serious ^i^ | not serious | none | 1145 cases 5165 controls | | **OR 0.93** (0.81 to 1.09) | - | ⨁◯◯◯ VERY LOW | CRITICAL |
|  |  |  |  |  |  |  | - | 0.0% |  | **--** |  |  |
| **QALYs** | | | | | | | | | | | | |
| 3 ^2,9,10,j^ | modelling studies | not serious | not serious | serious ^f^ | not serious | none | 4,400 to 7,100 | 3,900 to 5,000 | **Ratio 1**.12 to **1.44** | **500 to 2,100 more per 100.000** | ⨁◯◯◯ VERY LOW | CRITICAL |
| **Interval cancer** | | | | | | | | | | | | |
| 1 ^5,k,l^ | observational studies | serious ^h^ | not serious | serious ^m^ | not serious | none | Annual: 22% (21% to 30%) Biennial 27% (26% to 29%). Difference: 5,000 fewer per 100,000 breast cancers. | | | | ⨁◯◯◯ VERY LOW | CRITICAL |
| **Overdiagnosis** | | | | | | | | | | | | |
| 1 ^2,j^ | modelling studies | not serious | not serious | serious ^f^ | not serious | none | 904 | 609 | **Ratio 1.48** | **295 more per 100.000** | ⨁◯◯◯ VERY LOW | CRITICAL |
| **False positive results -10 year probability** | | | | | | | | | | | | |
| 1 ^6^ | observational studies | serious ^n^ | not serious | serious ^i^ | not serious | none | Annual 54% (53% to 55%) Biennial 34% (34% to 35%) Difference: 20,000 more per 100,000 exams | | | | ⨁◯◯◯ VERY LOW | CRITICAL |
| **False positive biopsy recommendation -10 year probability** | | | | | | | | | | | | |
| 1 ^6^ | observational studies | serious ^n^ | not serious | serious ^i^ | not serious | none | Annual 8% (7% to 9%) Biennial 5% (4% to 5%). Difference: 3,000 more per 100,000 exams. | | | | ⨁◯◯◯ VERY LOW | CRITICAL |
| **Radiation induce breast cancer** | | | | | | | | | | | | |
| 2 ^7,8,j^ | modelling studies | serious ^d,e^ | not serious | very serious ^f,g^ | not serious | none | 27 to 49 | 14 to 27 | **Ratio**  **1.81 to 1.93** | **13 more to 22 more per 100.000** | ⨁◯◯◯ VERY LOW | CRITICAL |
| **Death by radiation induced breast cancer** | | | | | | | | | | | | |
| 2 ^7,8,j^ | modelling studies | serious ^d,e^ | not serious | very serious ^f,g^ | not serious | none | 3 to 7 | 2 to 4 | **Ratio** **1.50 to 1.75** | **1 more to 3 more per 100.000** | ⨁◯◯◯ VERY LOW | CRITICAL |
| **Incidence of advanced cancer following first round of screening - not reported** | | | | | | | | | | | | |
| - | - | - | - | - | - | - | - | - | - | - | - | CRITICAL |

**CI:** Confidence interval; **OR:** Odds ratio. For modeling studies, certainty of evidence starts from low certainty and when there is more than one study informing an outcome, the number represents the range of point estimates reported across studies

#### Explanations

a. A systematic review comparing indirectly effects of different screening intervals vs. no screening showed the following results: screening interval <24 mo (5 studies), RR 0.82 (95%CI 0.72-0.94); and screening interval >=24 mo (3 studies), RR 1.04 (95%CI 0.72-1.50) (Canadian Task Force on Preventive Health Care).

b. Results based on registries from two time periods, before and after the modification of recommended screening interval from annual to biennial in women 50 years or older. Not all the participants of the screening program were adherent to the modification. Secular trend is plausible.

c. Modelling studies used different number of women screened for calculations: 1,000 in 1 study, 100,000 in 2 studies.

d. One or more studies did not report information about external validation for the estimated parameter of the models.

e. One or more studies did not report information sensitivity analysis for the estimated parameter of the models.

f. The comparison for any interval in the models was a no screening scenario. No direct comparisons were reported.

g. Models were constructed using data from surveillance registries of different countries, only one or no study used European data.

h. Intervals were classified based on the month ranges elapsed between two screening mammograms prior to diagnosis. Potential high risk of misclassification.

i. Results were extracted from groups of women with selected characteristics (e.g. normal weight, fatty or scattered fibroglandular breast density, or white race).

j. Modelling studies used 100,000 women screened for calculations.

k. In the Swedish two county trial with an average screening interval of 24 months, the calculated interval cancers for >0 to <12 months was 38%, and for 12 to <24 months was 68% (Tabar 1987).

l. Percentages are calculated based on the number of interval cancers over all breast cancers diagnosed in a certain period. Absolute differences are expressed over the number of detected breast cancers.

m. Based on breast cancer series from surveillance registries in the United States. Results were extracted from broader age groups (i.e- 40 to 85 years)-.

n. No clear information of how the intervals were estimated for the false positive cohorts or the number of individuals per interval.

#### References

1. Coldman, AJ. Impact of changing from annual to biennial mammographic screening on breast cancer outcomes in women aged 50-79 in British Columbia. J Med Screen; 2008.

2. Vilaprinyo, E. Cost-effectiveness and harm-benefit analyses of risk-based screening strategies for breast cancer. PLoS One; 2014.

3. Tsunematsu M, Kakehashi M.. An analysis of mass screening strategies using a mathematical model: comparison of breast cancer screening in Japan and the United States. J Epidemiol; 2015.

4. Yaffe, MJ. Clinical outcomes of modelling mammography screening strategies. Health Rep; 2015.

5. Miglioretti DL, Zhu W,Kerlikowske K,Sprague BL,Onega T,Buist DS,Henderson LM,Smith RA, Consortium., Breast,Cancer,Surveillance. Breast tumor prognostic characteristics and biennial vs annual mammography, age, and menopausal status. JAMA Oncol; 2015.

6. Dittus, K. Impact of mammography screening interval on breast cancer diagnosis by menopausal status and BMI. J Gen Intern Med ;

7. Yaffe, M.J. Risk of radiation-induced breast cancer from mammographic screening. Radiology; 2011.

8. Miglioretti, J. Radiation-induced breast cancer incidence and mortality from digital mammography screening.. Ann Intern Med; 2016.

9. Mittmann N. Cost-effectiveness of mammography from a publicly funded health care system perspective. CMAJ Open. 2018.

10. Arnold M. Is risk-stratified breast cancer screening economically efficient in Germany? PLoS One. 2019.

**Table S9. Question:** Triennial mammography screening compared to biennial mammography screening for early detection of breast cancer in women aged 50 to 69

| **Certainty assessment** | | | | | | | **№ of patients** | | **Effect** | | **Certainty** | **Importance** |
| --- | --- | --- | --- | --- | --- | --- | --- | --- | --- | --- | --- | --- |
| **№ of studies** | **Study design** | **Risk of bias** | **Inconsistency** | **Indirectness** | **Imprecision** | **Other considerations** | **triennial mammography screening** | **biennial mammography screening** | **Relative (95% CI)** | **Absolute (95% CI)** |  |  |
| **Breast cancer death averted** | | | | | | | | | | | | |
| 2 ^1,2,a,b^ | modelling studies | not serious ^c,d^ | not serious | serious ^e,f^ | not serious | none | 397 to 400 | 426 to 520 | **Ratio** **0.77 to 0.93** | **120 fewer to 29 fewer per 100,000** | ⨁◯◯◯ VERY LOW | CRITICAL |
| **QALYs** | | | | | | | | | | | | |
| 3 ^2,5,6,g^ | modelling studies | not serious | not serious | serious ^e^ | not serious | none | 3,300 to 4,386 | 3,900 to 5,000 | **Ratio 0.85 to 0.93** | **1,200 fewer to 328 fewer per 100,000** | ⨁◯◯◯ VERY LOW | CRITICAL |
| **Stage of breast cancer (IIB-IV)** | | | | | | | | | | | | |
| 1 ^4^ | observational studies | serious ^h^ | not serious | serious ^i^ | not serious | none | 468 cases 2415 controls | | **OR** 0.83 (0.65 to 1.07) | - | ⨁◯◯◯ VERY LOW | CRITICAL |
|  |  |  |  |  |  |  | - | 0.0% |  | **--** |  |  |
| **Interval cancer** | | | | | | | | | | | | |
| 1 ^3,j,k,l^ | observational studies | serious ^h^ | not serious | serious ^m^ | not serious | none | Reported estimates: triennial: 44% (41% to 48%), biennial: 41% (39% to 42%). Difference: 3,000 more per 100,000 breast cancers. | | | | ⨁◯◯◯ VERY LOW | CRITICAL |
| **Overdiagnosis** | | | | | | | | | | | | |
| 1 ^2,g^ | modelling studies | not serious | not serious | serious ^e^ | not serious | none | 500 | 609 | **Ratio 0.82** | **109 fewer per 100,000** | ⨁◯◯◯ VERY LOW | CRITICAL |
| **False positive results -10 year probability** | | | | | | | | | | | | |
| 1 ^4^ | observational studies | serious ^n^ | not serious | serious ^i^ | not serious | none | Triennial 25% (25% to 25%) Biennial 35% (35% to 36%) Difference: 10,000 fewer per 100,000 exams | | | | ⨁◯◯◯ VERY LOW | CRITICAL |
| **False positive biopsy recommendation -10 year probability** | | | | | | | | | | | | |
| 1 ^4^ | observational studies | serious ^n^ | not serious | serious ^i^ | not serious | none | Triennial 4% (4% to 4%) Biennial 5% (5% to 6%) Difference: 1,000 more per 100,000 exams. | | | | ⨁◯◯◯ VERY LOW | CRITICAL |
| **Incidence of advanced cancer following first round of screening - not reported** | | | | | | | | | | | | |
| - | - | - | - | - | - | - | - | - | - | - | - | CRITICAL |
| **Radiation adverse effects - not reported** | | | | | | | | | | | | |
| - | - | - | - | - | - | - | - | - | - | - | - | CRITICAL |

**CI:** Confidence interval; **OR:** Odds ratio. For modeling studies, certainty of evidence starts from low certainty and when there is more than one study informing an outcome, the number represents the range of point estimates reported across studies

#### Explanations

a. A systematic review comparing indirectly effects of different screening intervals vs. no screening showed the following results: screening interval <24 mo (5 studies), RR 0.82 (95%CI 0.72-0.94); and screening interval >=24 mo (3 studies), RR 1.04 (95%CI 0.72-1.50) (Canadian Task Force on Preventive Health Care).

b. Modelling studies, used different number of women screened for calculations: 1,000 in 1 studies, and 100,000 in 1 studies.

c. One or more studies did not report information about external validation for the estimated parameter of the models

d. One or more studies did not report information sensitivity analysis for the estimated parameter of the models

e. The comparison for any interval in the models was a no screening scenario. No direct comparisons were reported.

f. Models were constructed using data from surveillance registries of different countries, only one study or none used European population data.

g. Modelling study used 100,000 women screened for calculation.

h. Intervals were classified based on the month ranges elapsed between two screening mammograms prior to diagnosis. Potential high risk of misclassification.

i. Results were extracted from groups of women with selected characteristics (e.g. normal weight, fatty or scattered fibroglandular breast density, or white race) and/or broader age group (i.e- 50 to 74 years)-.

j. In the Swedish two county trial with an average screening interval of 24 months, the calculated interval cancers for >0 to <12 months was 38%, and for 12 to <24 months was 68% (Tabar 1987).

k. U.K. NHS Breast Screening Program (triennial screening program): in women aged 50-64 years the rate of interval cancer per 1,000 was 0.55 for <12 mo, 1.13 for 24 to <36 mo and 1.22 for 24 to <36 mo.

l. Percentages are calculated based on the number of interval cancers over all breast cancers diagnosed in a certain period. Absolute differences are expressed over the number of detected breast cancers.

m. Based on breast cancer series from surveillance registries in the United States.

n. No clear information of how the intervals were estimated for the false positive cohorts or the number of individuals per interval

#### References

1. Yaffe, MJ. Clinical outcomes of modelling mammography screening strategies. Health Rep; 2015.

2. Vilaprinyo, E. Cost-effectiveness and harm-benefit analyses of risk-based screening strategies for breast cancer. PLoS One; 2014.

3. Kerlikowske K, Zhu W,Hubbard RA,Geller B,Dittus K,Braithwaite D,Wernli KJ,Miglioretti DL,O'Meara ES, Consortium., Breast,Cancer,Surveillance. Outcomes of screening mammography by frequency, breast density, and postmenopausal hormone therapy. JAMA Intern Med; 2013.

4. O'Meara ES, Zhu W,Hubbard RA,Braithwaite D,Kerlikowske K,Dittus KL,Geller B,Wernli KJ,Miglioretti DL.. Mammographic screening interval in relation to tumour characteristics and false-positive risk by race/ethnicity and age. Cancer; 2013.

5. Mittmann N. Cost-effectiveness of mammography from a publicly funded health care system perspective. CMAJ Open. 2018.

6. Arnold M. Is risk-stratified breast cancer screening economically efficient in Germany? PLoS One. 2019.

**Table S10. Question:** Annual compared to triennial mammography for women 50-69 years

| **Certainty assessment** | | | | | | | **№ of patients** | | **Effect** | | **Certainty** | **Importance** |
| --- | --- | --- | --- | --- | --- | --- | --- | --- | --- | --- | --- | --- |
| **№ of studies** | **Study design** | **Risk of bias** | **Inconsistency** | **Indirectness** | **Imprecision** | **Other considerations** | **annual mammography screening** | **triennial mammography screening** | **Relative (95% CI)** | **Absolute (95% CI)** |
| **Breast cancer mortality** | | | | | | | | | | | | |
| 1 ^1,2,a^ | randomised trials | not serious | not serious | not serious | serious ^b^ | none | 209/37530 (0.6%) | 231/38429 (0.6%) | **RR 0.93** (0.76 to 1.12) | **42 fewer per 100.000** (from 144 fewer to 72 more) | ⨁⨁⨁◯ MODERATE | CRITICAL |
| **Breast cancer death averted** | | | | | | | | | | | | |
| 2 ^3,4,a,c^ | modelling studies | not serious ^d,e^ | not serious | serious ^f,g^ | not serious | none | 631 to 740 | 397 to 400 | **Ratio**  **1.59 to 1.85** | **234 more to 340 more per 100.000** | ⨁◯◯◯ VERY LOW | CRITICAL |
| **QALYs** | | | | | | | | | | | | |
| 3 ^4,7,8,h^ | modelling studies | not serious | not serious | serious ^f^ | not serious | none | 4,400 to 7,100 | 3,300 to 4,386 | **Ratio 1.33 to** **1.55** | **1,100 more to 3,100 more per 100.000** | ⨁◯◯◯ VERY LOW | CRITICAL |
| **Interval cancer** | | | | | | | | | | | | |
| 1 ^5,i,j,k^ | observational studies | serious ^l^ | not serious | serious ^m^ | not serious | none | Annual: 30% (29% to 31%) Triennial: 44% (41% to 48%) Difference: 14,000 fewer per 100,000 breast cancers. | | | | ⨁◯◯◯ VERY LOW | CRITICAL |
| **Overdiagnosis** | | | | | | | | | | | | |
| 1 ^4,h^ | modelling studies | not serious | not serious | serious ^f^ | not serious | none | 904 | 500 | **Ratio 1.81** | **404 more per 100.000** | ⨁◯◯◯ VERY LOW | CRITICAL |
| **False positive results -10 year probability** | | | | | | | | | | | | |
| 1 ^6^ | observational studies | serious ^n^ | not serious | serious ^o^ | not serious | none | Annual 55% (55% to 56%) Triennial 25% (25% to 25%) Difference: 30,000 more per 100,000 | | | | ⨁◯◯◯ VERY LOW | CRITICAL |
| **False positive biopsy recommendation -10 year probability** | | | | | | | | | | | | |
| 1 ^6^ | observational studies | serious ^n^ | not serious | serious ^o^ | not serious | none | Annual 10% (9% to 10%) Triennial 4% (4% to 4%) Difference: 6,000 more per 100,000. | | | | ⨁◯◯◯ VERY LOW | CRITICAL |
| **Stage of breast cancer (IIB-IV) - not reported** | | | | | | | | | | | | |
| - | - | - | - | - | - | - | - | - | - | - | - | CRITICAL |
| **Incidence of advanced cancer following first round of screening - not reported** | | | | | | | | | | | | |
| - | - | - | - | - | - | - | - | - | - | - | - | CRITICAL |
| **Radiation adverse effects - not reported** | | | | | | | | | | | | |
| - | - | - | - | - | - | - | - | - | - | - | - | CRITICAL |

**CI:** Confidence interval; **RR:** Risk ratio. For modeling studies, certainty of evidence starts from low certainty and when there is more than one study informing an outcome, the number represents the range of point estimates reported across studies

#### Explanations

a. A systematic review comparing indirectly effects of different screening intervals vs. no screening showed the following results: screening interval <24 mo (5 studies), RR 0.82 (95%CI 0.72-0.94); and screening interval >=24 mo (3 studies), RR 1.04 (95%CI 0.72-1.50) (Canadian Task Force on Preventive Health Care).

b. Wide confidence interval of effect

c. Modelling studies, used different number of women screened for calculations: 1,000 in 1 studies, and 100,000 in 1 studies.

d. One or more studies did not report information about external validation for the estimated parameter of the models

e. One or more studies did not report information sensitivity analysis for the estimated parameter of the models

f. The comparison for any interval in the models was a no screening scenario. No direct comparisons were reported.

g. Models were constructed using data from surveillance registries of different countries, only one study or none used European population data.

h. Modelling study used 100,000 women screened for calculation.

i. In the Swedish two county trial with an average screening interval of 24 months, the calculated interval cancers for >0 to <12 months was 38%, and for 12 to <24 months was 68% (Tabar 1987).

j. U.K. NHS Breast Screening Program (triennial screening program): in women aged 50-64 years the rate of interval cancer per 1,000 was 0.55 for <12 mo, 1.13 for 24 to <36 mo and 1.22 for 24 to <36 mo.

k. Percentages are calculated based on the number of interval cancers over all breast cancers diagnosed in a certain period. Absolute differences are expressed over the number of detected breast cancers.

l. Intervals were classified based on the month ranges elapsed between two screening mammograms prior to diagnosis. Potential high risk of misclassification.

m. Based on breast cancer series from surveillance registries in the United States. Results were extracted from broader age group (i.e- 40 to 74 years)-.

n. No clear information of how the intervals were estimated for the false positive cohorts or the number of individuals per interval

o. Results were extracted from groups of women with selected characteristics (e.g. normal weight, fatty or scattered fibroglandular breast density, or white race) and/or broader age group (i.e- 50 to 74 years)-. .

#### References

1. Duffy, SW. Long term mortality results from the UK screening frequency trial in 6th European reast Cancer Conference: Berlin, Germany.

2. Breast Screening Frequency Trial, Group. The frequency of breast cancer screening: results from the UKCCCR Randomised Trial. United Kingdom Co-ordinating Committee on Cancer Research. Eur J Cancer; 2002.

3. Yaffe, MJ. Clinical outcomes of modelling mammography screening strategies. Health Rep; 2015.

4. Vilaprinyo, E. Cost-effectiveness and harm-benefit analyses of risk-based screening strategies for breast cancer. PLoS One; 2014.

5. Kerlikowske K, Zhu W,Hubbard RA,Geller B,Dittus K,Braithwaite D,Wernli KJ,Miglioretti DL,O'Meara ES, Consortium., Breast,Cancer,Surveillance. Outcomes of screening mammography by frequency, breast density, and postmenopausal hormone therapy. JAMA Intern Med; 2013.

6. O'Meara ES, Zhu W,Hubbard RA,Braithwaite D,Kerlikowske K,Dittus KL,Geller B,Wernli KJ,Miglioretti DL.. Mammographic screening interval in relation to tumour characteristics and false-positive risk by race/ethnicity and age. Cancer; 2013.

7. Mittmann N. Cost-effectiveness of mammography from a publicly funded health care system perspective. CMAJ Open. 2018.

8. Arnold M. Is risk-stratified breast cancer screening economically efficient in Germany? PLoS One. 2019.

**Table S11. Question:** annual mammography screening compared to biennial mammography screening for women 70 to 74

| **Certainty assessment** | | | | | | | **№ of patients** | | **Effect** | | **Certainty** | **Importance** |
| --- | --- | --- | --- | --- | --- | --- | --- | --- | --- | --- | --- | --- |
| **№ of studies** | **Study design** | **Risk of bias** | **Inconsistency** | **Indirectness** | **Imprecision** | **Other considerations** | **annual mammography screening** | **biennial mammography screening** | **Relative (95% CI)** | **Absolute (95% CI)** |  |  |
| **Breast cancer death averted** | | | | | | | | | | | | |
| 2 ^1,2,a^ | modelling studies | not serious ^b,c^ | not serious | serious ^d,e^ | not serious | none | 100 to 142 | 90 to 145 | **Ratio**  **0.98 to 1.11** | **3 fewer to 10 more per 100,00** | ⨁◯◯◯ VERY LOW | CRITICAL |
| **Stage of breast cancer (IIB-IV)** | | | | | | | | | | | | |
| 1 ^3^ | observational studies | serious ^f^ | serious | serious ^g^ | not serious | none | 254 cases 2910 controls | | **OR 0.98** (0.76 to 1.27) | - | ⨁◯◯◯ VERY LOW | CRITICAL |
|  |  |  |  |  |  |  | - | 0.0% |  | **-- per 100.000** (from -- to --) |  |  |
| **Interval cancer** | | | | | | | | | | | | |
| 1 ^4,h^ | observational studies | serious ^f^ | not serious | serious ^i^ | not serious | none | Annual: 23% (22% to 25%) Biennial: 33% (30% to 36%) Difference: 10,000 fewer per 100,000 breast cancers. | | | | ⨁◯◯◯ VERY LOW | CRITICAL |
| **Overdiagnosis** | | | | | | | | | | | | |
| 1 ^2,j^ | modelling studies | not serious | not serious | serious ^d,e^ | not serious | none | 269 | 236 | **Ratio 1.14** | **33 more per 100.000** | ⨁◯◯◯ VERY LOW | CRITICAL |
| **QALYs** | | | | | | | | | | | | |
| 2 ^1,2,j^ | modelling studies | not serious | not serious | serious ^d,e^ | not serious | none | 336 to 600 | 427 to 500 | **Ratio 0.79 to 1.20** | **91 fewer to 100 more per 100.000** | ⨁◯◯◯ VERY LOW | CRITICAL |
| **False positive - 10 year probability** | | | | | | | | | | | | |
| 1 ^4^ | observational studies | serious ^k^ | not serious | not serious | not serious | none | Annual 47% (45% to 50%), Biennial 27% (26% to 28%). Difference: 20,000 more per 100,000 exams. | | | | ⨁◯◯◯ VERY LOW | CRITICAL |
| **False positive biopsy recommendation - 10 year probability** | | | | | | | | | | | | |
| 1 ^4^ | observational studies | serious ^k^ | not serious | not serious | not serious | none | Annual 9% (8% to 11%), Biennial 4% (4% to 5%) . Difference: 5,000 more per 100,000 exams. | | | | ⨁◯◯◯ VERY LOW | CRITICAL |
| **Incidence of advanced cancer following first round of screening - not reported** | | | | | | | | | | | | |
| - | - | - | - | - | - | - | - | - | - | - | - | CRITICAL |
| **Radiation adverse effects - not reported** | | | | | | | | | | | | |
| - | - | - | - | - | - | - | - | - | - | - | - | CRITICAL |

**CI:** Confidence interval; **OR:** Odds ratio. For modeling studies, certainty of evidence starts from low certainty and when there is more than one study informing an outcome, the number represents the range of point estimates reported across studies

#### Explanations

a. Modelling studies, used different number of women screened for calculations: 1,000 in 1 study, and 100,000 in 1 study.

b. One or more studies did not report information about external validation for the estimated parameter of the models.

c. One or more studies did not report information sensitivity analysis for the estimated parameter of the models.

d. The comparison for any interval in the models was a no screening scenario. No direct comparisons were reported.

e. Reports did not include the age range 70-74. Results were calculated by subtracting the effects of overlapping age groups.

f. Intervals were classified based on the month ranges elapsed between two screening mammograms prior to diagnosis. Potential high risk of misclassification.

g. Results were extracted from groups of women with selected characteristics (e.g. normal weight, fatty or scattered fibroglandular breast density, or white race).

h. Percentages are calculated based on the number of interval cancers over all breast cancers diagnosed in a certain period. Absolute differences are expressed over the number of detected breast cancers.

i. Based on breast cancer series from surveillance registries in the United States. Results were extracted from broader age group (i.e- 66 to 89 years)-.

j. Modelling study, used 100,000 women screened for calculations.

k. No clear information of how the intervals were estimated for the false positive cohorts or the number of individuals per interval.

#### References

1. Yaffe, MJ. Clinical outcomes of modelling mammography screening strategies. Health Rep; 2015.

2. Vilaprinyo, E. Cost-effectiveness and harm-benefit analyses of risk-based screening strategies for breast cancer. PLoS One; 2014.

3. Miglioretti DL, Zhu W,Kerlikowske K,Sprague BL,Onega T,Buist DS,Henderson LM,Smith RA, Consortium., Breast,Cancer,Surveillance. Breast tumor prognostic characteristics and biennial vs annual mammography, age, and menopausal status. JAMA Oncol; 2015.

4. Braithwaite D, Zhu W,Hubbard RA,O'Meara ES,Miglioretti DL,Geller B,Dittus K,Moore D,Wernli KJ,Mandelblatt J,Kerlikowske K, Consortium., Breast,Cancer,Surveillance. Screening outcomes in older US women undergoing multiple mammograms in community practice: does interval, age, or comorbidity score affect tumour characteristics or false positive rates?. J Natl Cancer Inst; 2013.

**Table S12. Question:** Triennial mammography screening compared to biennial mammography screening for early detection of breast cancer in women aged 70 to 74

| **Certainty assessment** | | | | | | | **№ of patients** | | **Effect** | | **Certainty** | **Importance** |
| --- | --- | --- | --- | --- | --- | --- | --- | --- | --- | --- | --- | --- |
| **№ of studies** | **Study design** | **Risk of bias** | **Inconsistency** | **Indirectness** | **Imprecision** | **Other considerations** | **triennial mammography screening** | **biennial mammography screening** | **Relative (95% CI)** | **Absolute (95% CI)** |
| **Breast cancer death averted** | | | | | | | | | | | | |
| 2 ^1,2,a^ | modelling studies | not serious ^b,c^ | not serious | serious ^d,e^ | not serious | none | 80 to 136 | 90 to 145 | **Ratio**  **0.89 to 0.94** | **10 fewer to 9 fewer per 100.000** | ⨁◯◯◯ VERY LOW | CRITICAL |
| **QALYs** | | | | | | | | | | | | |
| 2 ^1,2,f^ | modelling studies | not serious | not serious | serious ^d,e^ | not serious | none | 300 to 398 | 427 to 500 | **Ratio 0.60 to** **0.93** | **200 fewer to 29 fewer per 100.000** | ⨁◯◯◯ VERY LOW | CRITICAL |
| **Overdiagnosis** | | | | | | | | | | | | |
| 1 ^2,f^ | modelling studies | not serious | not serious | serious ^d,e^ | not serious | none | 193 | 236 | **Ratio 0.82** | **43 fewer per 100.000** | ⨁◯◯◯ VERY LOW | CRITICAL |
| **False positive** | | | | | | | | | | | | |
| 2 ^1,2,a^ | modelling studies | not serious ^b,c^ | not serious | serious ^d,e^ | not serious | none | 2,295 to 12,700 | 3,495 to 17,400 | **Ratio**  **0.66 to 0.73** | **from 4700 fewer to 1164 fewer per 100.000** | ⨁◯◯◯ VERY LOW | CRITICAL |
| **False positive biopsies** | | | | | | | | | | | | |
| 2 ^1,2,a^ | modelling studies | not serious ^b,c^ | not serious | serious ^d,e^ | not serious | none | 171 to 3,200 | 287 to 3,500 | **Ratio**  **0.59 to 0.91** | **from 300 fewer to 116 fewer per 100.000** | ⨁◯◯◯ VERY LOW | CRITICAL |
| **Stage of breast cancer (IIB-IV) - not reported** | | | | | | | | | | | | |
| - | - | - | - | - | - | - | - | - | - | - | - | CRITICAL |
| **QALY - not reported** | | | | | | | | | | | | |
| - | - | - | - | - | - | - | - | - | - | - | - | CRITICAL |
| **Interval cancer - not reported** | | | | | | | | | | | | |
| - | - | - | - | - | - | - | - | - | - | - | - | CRITICAL |
| **Incidence of advanced cancer following first round of screening - not reported** | | | | | | | | | | | | |
| - | - | - | - | - | - | - | - | - | - | - | - | CRITICAL |
| **False positive adverse effects - not reported** | | | | | | | | | | | | |
| - | - | - | - | - | - | - | - | - | - | - | - | CRITICAL |
| **Overdiagnosis - not reported** | | | | | | | | | | | | |
| - | - | - | - | - | - | - | - | - | - | - | - | CRITICAL |
| **Radiation adverse effects - not reported** | | | | | | | | | | | | |
| - | - | - | - | - | - | - | - | - | - | - | - | CRITICAL |

**CI:** Confidence interval. For modeling studies, certainty of evidence starts from low certainty and when there is more than one study informing an outcome, the number represents the range of point estimates reported across studies

#### Explanations

a. Modelling studies, used different number of women screened for calculations: 1,000 in 1 study, and 100,000 in 1 study.

b. One or more studies did not report sensitivity analysis information for the estimated parameters of the models.

c. One or more studies did not report information about external validation for the estimated parameters of the models.

d. The comparison for any interval in the models was a no screening scenario. No direct comparisons were reported.

e. Reports did not include the age group 70-74. Results were calculated by subtracting the effects of overlapping age groups.

f. Modelling study, used 100,000 women screened for calculations.

#### References

1. Yaffe, MJ. Clinical outcomes of modelling mammography screening strategies. Health Rep; 2015.

2. Vilaprinyo, E. Cost-effectiveness and harm-benefit analyses of risk-based screening strategies for breast cancer. PLoS One; 2014.

**Table S13. Question:** Annual mammography screening compared to triennial mammography screening for women 70 to 74

| **Certainty assessment** | | | | | | | **№ of patients** | | **Effect** | | **Certainty** | **Importance** |
| --- | --- | --- | --- | --- | --- | --- | --- | --- | --- | --- | --- | --- |
| **№ of studies** | **Study design** | **Risk of bias** | **Inconsistency** | **Indirectness** | **Imprecision** | **Other considerations** | **annual mammography screening** | **triennial mammography screening** | **Relative (95% CI)** | **Absolute (95% CI)** |
| **Breast cancer death averted** | | | | | | | | | | | | |
| 2 ^1,2,a^ | modelling studies | not serious ^b,c^ | not serious | serious ^d,e^ | not serious | none | 100 to 142 | 80 to 136 | **Ratio 1.04 to 1.25** | **6 more to 20 more per 100.000** | ⨁◯◯◯ VERY LOW | CRITICAL |
| **QALYs** | | | | | | | | | | | | |
| 1 ^2,f^ | observational studies | not serious | not serious | serious ^d,e^ | not serious | none | 336 to 600 | 300 to 398 | **Ratio 0.84** | **62 fewer to 300 more per 100.000** | ⨁◯◯◯ VERY LOW | CRITICAL |
| **Overdiagnosis** | | | | | | | | | | | | |
| 1 ^2,f^ | modelling studies | not serious | not serious | serious ^d,e^ | not serious | none | 269 | 193 | **Ratio 1.39** | **76 more per 100.000** | ⨁◯◯◯ VERY LOW | CRITICAL |
| **False positive** | | | | | | | | | | | | |
| 2 ^1,2,a^ | modelling studies | not serious ^b,c^ | not serious | serious ^d,e^ | not serious | none | 5,766 to 24,500 | 2,295 to 12,700 | **Ratio**  **1.93 to 2.51** | **3,471 more to 11,800 more per 100.000** | ⨁◯◯◯ VERY LOW | CRITICAL |
| **False positive biopsies** | | | | | | | | | | | | |
| 2 ^1,2,a^ | modelling studies | not serious ^b,c^ | not serious | serious ^d,e^ | not serious | none | 428 to 3,200 | 171 to 3,200 | **Ratio**  **1.0 to 2.5** | **0 more to 257 more per 100.000** | ⨁◯◯◯ VERY LOW | CRITICAL |
| **Stage of breast cancer (IIB-IV) - not reported** | | | | | | | | | | | | |
| - | - | - | - | - | - | - | - | - | - | - | - | CRITICAL |
| **QALY - not reported** | | | | | | | | | | | | |
| - | - | - | - | - | - | - | - | - | - | - | - | CRITICAL |
| **Interval cancer - not reported** | | | | | | | | | | | | |
| - | - | - | - | - | - | - | - | - | - | - | - | CRITICAL |
| **Incidence of advanced cancer following first round of screening - not reported** | | | | | | | | | | | | |
| - | - | - | - | - | - | - | - | - | - | - | - | CRITICAL |
| **False positive adverse effects - not reported** | | | | | | | | | | | | |
| - | - | - | - | - | - | - | - | - | - | - | - | CRITICAL |
| **Overdiagnosis - not reported** | | | | | | | | | | | | |
| - | - | - | - | - | - | - | - | - | - | - | - | CRITICAL |
| **Radiation adverse effects - not reported** | | | | | | | | | | | | |
| - | - | - | - | - | - | - | - | - | - | - | - | CRITICAL |

**CI:** Confidence interval. For modeling studies, certainty of evidence starts from low certainty and when there is more than one study informing an outcome, the number represents the range of point estimates reported across studies

#### Explanations

a. Modelling studies, used different number of women screened for calculations: 1,000 in 1 study, and 100,000 in 1 study.

b. One or more studies did not report sensitivity analysis information for the estimated parameters of the models.

c. One or more studies did not report information about external validation for the estimated parameters of the models.

d. The comparison for any interval in the models was a no screening scenario. No direct comparisons were reported.

e. Reports did not include the age group 70-74. Results were calculated by subtracting the effects of overlapping age groups.

f. Modelling study, used 100,000 women screened for calculations.

#### References

1. Yaffe, MJ. Clinical outcomes of modelling mammography screening strategies. Health Rep; 2015.

2. Vilaprinyo, E. Cost-effectiveness and harm-benefit analyses of risk-based screening strategies for breast cancer. PLoS One; 2014
